# Insight into the template effect of vesicles on the laccase-catalyzed oligomerization of *N*-phenyl-1,4-phenylenediamine from Raman spectroscopy and cyclic voltammetry measurements

**DOI:** 10.1038/srep30724

**Published:** 2016-08-26

**Authors:** Aleksandra Janoševic Ležaić, Sandra Luginbühl, Danica Bajuk-Bogdanović, Igor Pašti, Reinhard Kissner, Boris Rakvin, Peter Walde, Gordana Ćirić-Marjanović

**Affiliations:** 1 University of Belgrade-Faculty of Pharmacy, Department of Physical Chemistry and Instrumental Methods, Vojvode Stepe 450, 11221 Belgrade, Serbia; 2 Department of Materials, ETH Zürich, Vladimir-Prelog-Weg 5, CH-8093 Zürich, Switzerland; 3 Faculty of Physical Chemistry, University of Belgrade, Studentski trg 12-16, 11158 Belgrade, Serbia; 4 Department of Chemistry and Applied Biosciences, ETH Zürich, Vladimir-Prelog-Weg 2, CH-8093 Zürich, Switzerland; 5 Division of Physical Chemistry, Institute Ruđer Bošković, Bijenička cesta 54, HR-10000 Zagreb, Croatia

## Abstract

We report about the first Raman spectroscopy study of a vesicle-assisted enzyme-catalyzed oligomerization reaction. The aniline dimer *N*-phenyl-1,4-phenylenediamine (= *p*-aminodiphenylamine, PADPA) was oxidized and oligomerized with *Trametes versicolor* laccase and dissolved O_2_ in the presence of sodium bis(2-ethylhexyl)sulfosuccinate (AOT) vesicles (80–100 nm diameter) as templates. The conversion of PADPA into oligomeric products, poly(PADPA), was monitored during the reaction by *in situ* Raman spectroscopy. The results obtained are compared with UV/vis/NIR and EPR measurements. All three complementary methods indicate that at least some of the poly(PADPA) products, formed in the presence of AOT vesicles, resemble the conductive emeraldine salt form of polyaniline (PANI-ES). The Raman measurements also show that structural units different from those of “ordinary” PANI-ES are present too. Without vesicles PANI-ES-like products are not obtained. For the first time, the as-prepared stable poly(PADPA)-AOT vesicle suspension was used directly to coat electrodes (without product isolation) for investigating redox activities of poly(PADPA) by cyclic voltammetry (CV). CV showed that poly(PADPA) produced with vesicles is redox active not only at *pH* 1.1–as expected for PANI-ES–but also at *pH* 6.0, unlike PANI-ES and poly(PADPA) synthesized without vesicles. This extended *pH* range of the redox activity of poly(PADPA) is important for applications.

Recently, we have shown that *Trametes versicolor* laccase and O_2_ (TvL/O_2_) can efficiently oxidize the aniline dimer *N*-phenyl-1,4-phenylenediamine (=*p*-aminodiphenylamine, PADPA, Fig. 1) in the presence of 80–100 nm-sized sodium bis(2-ethylhexyl) sulfosuccinate (AOT) vesicles (Fig. 2), wherein the oxidized PADPA molecules subsequently react to form oligomeric or polymeric forms of PADPA, abbreviated as poly(PADPA)^1^. The reaction conditions were optimized in order to achieve a high PADPA conversion with a low enzyme concentration and avoiding product precipitation after the reaction had completed. In addition, the reaction conditions were tuned to obtain reaction products with a low absorption at ≈500 nm and high-intensity absorption bands at ≈400 and ≈1000 nm, which are characteristic of the electrically conductive polyaniline emeraldine salt (PANI-ES, Fig. 1)^2^,^3^,^4^,^5^. The best conditions found by applying the above criteria are as follows: [AOT] = 1.5 mM, [PADPA] = 1.0 mM, [TvL] ≈32 or 64 nM, in H_2_O with [H_2_PO_4_^−^ + H_3_PO_4_] = 0.1 M at *pH* = 3.5 and room temperature. With these conditions, the vesicular reaction suspension was found to be remarkably stable; no product precipitation occurred even after one month at room temperature. Consequently, the reaction can be followed and characterized *in situ* by UV/vis/NIR and EPR spectroscopy^1^. Furthermore, due to the high colloidal stability of the suspension, it can immediately be used–without any separation or modification steps−for subsequent applications, for example ink-jet printing^6^. This high stability in suspension is remarkable for a PANI-ES type poly(PADPA) product, as PANI-ES^7^,^8^,^9^,^10^ and poly(PADPA)^11^ synthesized by conventional chemical methods will precipitate during synthesis. Furthermore, the classical chemical synthesis of PANI-ES requires powerful oxidizing agents and a low *pH*, typically between 0 and 2^12^. In contrast, the enzymatic synthesis of the PANI-ES like product, poly(PADPA), is conducted under milder conditions (*pH* = 3.5) and by using an enzyme and O_2_ as the oxidizing agent, see also Shumakovich *et al*.^13^.

The reaction of PADPA with TvL/O_2_ leads to an oligo- or polymerization driven by radicals, whereby the first step of the reaction is most likely the formation of the radical cation, PADPA^•+ ^11,14. Two of these radical cations possibly directly combine and then react further, upon oxidation, with other PADPA radical cations, to finally form positively charged PADPA-chains with a high concentration of unpaired electrons in polaron form, typical for PANI-ES (Fig. 1)^1^,^11^,^13^. However, from previous studies it became evident that the products obtained from the oxidation of PADPA have a lower molar mass on average than conventional PANI-ES^1^,^8^,^15^. It seems that oligomeric rather than polymeric products are obtained from PADPA. Since the average degree of oligo-/polymerization is still not clear, and for the sake of convenience, we will continue using “poly(PADPA)” as general term for the oxidative reaction products obtained from PADPA.

Radical polymerizations involving radical–radical couplings of the type investigated here are difficult to control due to the high reactivity and low selectivity of the intermediate aromatic radical species. In this context the AOT vesicles have been found to be crucial for the TvL/O_2_-promoted oligo- or polymerization of PADPA: under the elaborated optimal conditions (see above), the vesicles direct the course of the reaction towards the desired poly(PADPA) end-products, *i.e.*, PANI-ES like molecules^1^. In particular, the soft surface of the vesicle membrane clearly exerts control over the oxidation and the polymerization steps of the reaction. Similar effects of soft interfaces as “reaction regulators” (templates) of enzyme-catalyzed oxidative polymerizations have been known for several years^16^,^17^,^18^,^19^. They have been found with anionic micelles, as another type of molecular assemblies^13^,^18^,^20^,^21^,^22^, or with anionic polymers, such as sulfonated polystyrene (SPS)^18^,^23^,^24^,^25^,^26^,^27^,^28^,^29^. For all these previous examples, the regulating effect of the templates is still not completely understood. Our long-term goal is to better understand the template effect observed with vesicles.

Herein, we applied for the first time Raman spectroscopy measurements of a vesicle-assisted enzyme-catalyzed oligomerization/polymerization reaction. We showed that Raman spectroscopy is a convenient and sensitive method for *in situ* monitoring of the oxidation and oligomerization/polymerization of PADPA with TvL/O_2_ in the presence of AOT vesicles, as well as in the absence of the vesicles. In contrast to the previously applied UV/vis/NIR and EPR spectroscopy measurements^1^, the present *in situ* Raman spectroscopy measurements of the reaction mixture during the course of reaction provide novel and detailed information on the evolution of the molecular structures of the oligomeric PADPA products through vibrational characteristics of distinct functional groups^4^,^30^ and through changes in their abundancies. This yields new insight into the effect of vesicles on the structural evolution of poly(PADPA).

An additional advantage of the *in situ* measurements is that possible structural changes in the products which may occur upon product isolation are avoided. For a comparison, the *in situ* Raman spectra of the final poly(PADPA) products synthesized with and without AOT vesicles are compared (i) with the corresponding Raman spectra of final *isolated solid* poly(PADPA) products, as well as (ii) with the Raman spectra reported in the literature for solid PANI salts and bases, produced by classical chemical polymerization.

Compared to our previous work^1^, we also carried out additional EPR measurements of the reaction mixture during the course of the reaction and made a more detailed analysis of the EPR spectra.

In addition to the EPR and Raman spectroscopy measurements, we present the first results of cyclic voltammetry (CV) measurements of this system, which demonstrate that the final products in the reaction suspension with vesicles are redox-active, as expected for PANI-ES-like molecules. Moreover, the redox activity of poly(PADPA) produced with vesicles is preserved at higher *pH* values compared to ordinary PANI-ES and poly(PADPA) produced enzymatically without vesicles. The vesicular reaction suspension was directly used for these electrochemical measurements, *i.e.* without any product isolation, which paves the way for further electrochemical applications of this type of AOT vesicle/PANI-ES-like system.

## Results and Discussion

### Reactions of PADPA with TvL/O_2_ Analyzed by UV/vis/NIR Spectroscopy

If an air-exposed aqueous suspension of large unilamellar AOT vesicles with diameters of about 80–100 nm is incubated at an AOT concentration of 1.5 mM in the *pH* = 3.5 solution (see Fig. 2 and *Methods*) and *T* ≈ 25 °C with 1.0 mM PADPA and ≈32 or 64 nM TvL, oxidation of PADPA and the formation of dark-green oligomeric and/or polymeric products without any visible precipitation is observed^1^. Since the reaction products remain finely dispersed throughout the entire course of the reaction with vesicles, UV/vis/NIR spectroscopy can be used to monitor the progress of the reaction by recording spectra of the entire reaction mixture as a function of reaction time^1^. Figure 3a shows the UV/vis/NIR spectroscopic changes occurring from the early stage up to 4 h of the reaction. The results presented here, with [TvL] = 64 nM and a reaction volume of 10 mL, are in good agreement with those obtained previously^1^ for a reaction mixture of the same composition, [TvL] = 32 nM, and a reaction volume of 15 mL, when both reactions are carried out in the *same* type of glass bottles (see *Methods*). This confirms the high reproducibility of the reaction, despite the heterogeneity of the system, and both used enzyme concentrations (32 or 64 nM) can be regarded as optimal. The products formed in the presence of the vesicular templates have a characteristic maximum absorbance at ≈1000 nm (A_≈1000_) and at ≈420 nm, with a minimum at ≈500 nm. Based on literature data^3^, the bands at ≈1000 nm and ≈420 nm correspond to π → polaron band and polaron band → π^*^ transitions of PANI and indicate the formation of the polaron form of PAN-ES-like poly(PADPA) chains. The band at 1000 nm is also indicative for *high delocalization* of polarons, *expanded coil conformation* of chains and consequently *high conductivity*^3^,^31^. Under the reaction conditions used, there is a continuous increase of A_≈1000_ with time until ≈3 h, after which there are no more significant changes in the absorption spectrum (Fig. 3a). If the reaction is carried out *without vesicles*, but otherwise identical composition (template - free system), the dark-green products are not formed, and precipitation begins after about 2–3 hours (Supplementary Fig. S1A)^1^. In the UV/vis/NIR spectra of this template-free system the characteristic band of delocalized polarons at ~1000 nm was not developed, but a band indicative for *localized* polarons is observed with a maximum at 700–780 nm^3^. Overall, it is very clear that the course of the reaction in the presence of AOT vesicles as well as the products obtained in the vesicular system are very different from the reaction and products of the template-free system.

### Reactions of PADPA with TvL/O_2_ Analysed by EPR Spectroscopy

In an extension of our previous EPR measurements^1^, the reaction mixture with AOT vesicles was analyzed by EPR spectroscopy in more detail during the first 2 h. The recorded EPR spectra are shown in Fig. 3b. All samples analyzed show the presence of unpaired electrons. At the very beginning of the reaction, after *t* = 1 min, the signal intensity is rather weak and the spectrum is very broad without any hyperfine structure. If the reaction is run without vesicles, a weak and broad signal is also observed after *t* = 1 min. In this case, however, the EPR spectrum shows a hyperfine coupling (Supplementary Fig. S1B). The calculated *g*-value of the radical species which are formed at the very beginning of the reaction in the absence of vesicles is *g* = 2.0031 ± 0.0001. Based on this *g*-value and the hyperfine splitting which is characteristic for arylamines^32^, we propose that the initial EPR signal is due to PADPA^•+^, which is formed at the initial stage of the reaction without as well as with vesicles. The reason that hyperfine coupling is not detected during the reaction with vesicles may be because binding of PADPA^•+^ to the vesicles leads to a broadening of the hyperfine signals. For reaction mixtures with vesicles in a time interval from 5 to 120 min, a sharp EPR signal of increasing intensity can be seen (Fig. 3b). All measured EPR spectra can be fitted by assuming the presence of two principal radical species (structural entities), one being the reaction intermediate formed initially (most likely PADPA^•+^), and the other being the main final reaction product. The *g*-value for the radical centers in this final reaction product was determined to be *g* = 2.0030 ± 0.0001. In Fig. 3c, the integral of the EPR spectra originating from the final reaction product obtained with the vesicles is plotted *vs.* reaction time and overlaid with A_1000_ *vs.* time as obtained from the UV/vis/NIR measurements. Note that the values for the integrals of the EPR spectra were scaled to match the course of the absorbance at 1000 nm. The time dependence of both signals is remarkably similar, confirming the correlation between A_≈1000_ and the presence of unpaired electrons in the product, as it is typical for molecules with structural units that resemble the conductive polaron state of PANI-ES. With the mentioned fitting, the changes of the relative abundance of the two radical species during the reaction in the presence of the vesicles were determined (Fig. 3d). After 120 min the initially formed radical species (most likely PADPA^•+^) disappeared, while the radical species of the (main) products clearly dominated. For the reaction without vesicles (template-free system), the total concentration of radicals never reached the levels of the reaction with vesicles^1^.

### Reactions of PADPA with TvL/O_2_ in the Presence of Vesicles Analysed by *in situ* Raman Spectroscopy Measurements

In order to gain information on chemical and structural changes of the reactive species during the enzymatic oxidative oligo- and polymerization of PADPA, and in order to gain new insights into the effect of vesicles, *in situ* Raman spectroscopy was applied as an alternative method to UV/vis/NIR and EPR spectroscopy.

Before measuring the reaction mixtures, reference spectra of the various reaction components (Milli-Q water, the phosphate solution, the AOT vesicle suspension, the PADPA solution, and the PADPA/AOT vesicle suspension) were recorded (Supplementary Fig. S2). Afterwards, *in situ* Raman spectra of the reaction mixture containing AOT vesicles and PADPA were measured at different times after initiating the reaction by adding TvL (Fig. 4). For this, small volumes were withdrawn from the reaction mixture at various reaction times and transferred into sample wells of the gold sample slide, see *Methods*. Immediately afterwards, the Raman spectrum of the reaction mixture droplet was measured. The observed Raman bands clearly originate mainly from the reaction intermediates and products. Contributions from AOT are negligible.

Although the interpretation of Raman spectra of PANI samples, with their various possible chemical structures, states of oxidation and degree of protonation is still in progress^33^, the assignments of the observed spectral changes are based on current knowledge elaborated for chemically synthesized poly(PADPA)^11^ and PANI^33^,^34^,^35^. Compared to the recent Raman analysis of thin PANI films formed on a gold surface^33^, where the authors found that the interaction of chemically adsorbed aniline oligomers (formed during the early stage of the reaction) with the smooth gold support causes enhancement and shifts of some peaks in the Raman spectra of PANI films, the *in situ* Raman spectra which were recorded here (Fig. 4) originate from the molecules present in the reaction mixture. There are no noticeable interactions between the gold support and the reaction mixture which would alter the spectrum (Supplementary Fig. S3), *i.e.,* the influence of the used sample support on the Raman spectra is negligible (Supplementary Figs S3 and S4).

In the following, the observed Raman bands are assigned and the meaning of their changes with time are discussed, listed according to the functional groups/structural units indicated to be present in reaction intermediates and final products.

#### Bands characteristic of quinonoid and semiquinonoid structures

**Figure 1:**
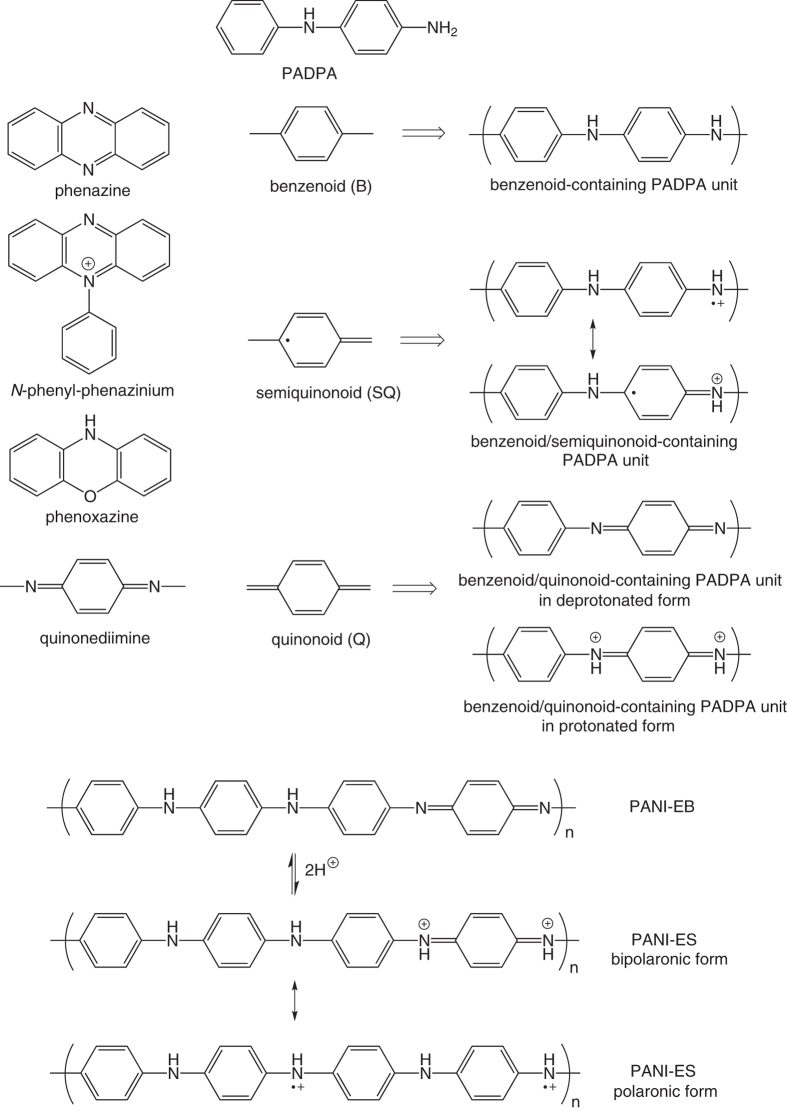
Chemical structure of *p*-aminodiphenylamine (PADPA, *N*-phenyl-1,4-phenylenediamine), the different poly(PADPA) structural units mentioned in the text, the emeraldine base form of polyaniline (PANI-EB), and the emeraldine salt form of polyaniline (PANI-ES).

Already 1 min after the addition of TvL, a strong band appears at 1596 cm^−1^ and remains a characteristic peak in all spectra recorded further on. This band is attributed to the C=C and C~C stretching vibrations of quinonoid (Q) and semiquinonoid (SQ) rings, *ν* (C=C)_Q_ and *ν* (C~C)_SQ_ (Fig. 1), where ″~″ denotes a bond intermediate between the single and double bond^11^,^34^,^35^.

The band at ≈1498 cm^−1^ (visible for *t* = 1–120 min) can be assigned to the C = N stretching in quinonediimine units, *ν* (C = N)_Q_ (Fig. 1)^34^,^35^. Furthermore, there is a weak band at ≈1505 cm^−1^ at *t* = 1 min, which later develops into a strong band at 1513–1520 cm^−1^. This band can be attributed to N–H bending vibrations, *δ* (N–H), and was previously correlated with SQ structures in PANI^34^,^35^. In the spectra taken at *t* = 1, 5, 10 and 15 min, the *ν* (C = N)_Q_ band is stronger than the *δ* (N–H) band, but the intensity of the *δ* (N–H) band gradually increases with time so that at *t* = 20 min the two bands have almost equal intensities. At *t* = 120 min the *δ* (N–H) band becomes more intense than the *ν* (C = N)_Q_ band, and for times after *t* > 120 min the peak at ≈1498 cm^−1^ disappears completely. This behavior indicates an increase of the protonation level and an increase in the content of SQ units in the reaction products with increasing reaction time.

The bands due to the C–H bending in-plane vibrations of SQ/Q, *δ* (C–H)_SQ,Q_, are observed at 1163–1168 cm^−1^.

#### Bands characteristic of polaron structures

The broad band assigned to C–N^+^ stretching vibrations, *ν* (C–N^+^), and attributable mainly to *ν* (C–N^•+^)_SQ_ vibration in polaron structures, with possible contribution of *ν* (C–N^+^) ring-stretching vibration in *N*-phenazine-type units^34^,^35^, is observed at ≈1350 cm^−1^ for *t* = 1 min and at ≈1376 cm^−1^ for t = 5 min. At the beginning of the reaction (*t* = 1 min) this band is rather weak, but its relative intensity increases with time. The red shift of the *ν* (C–N^•+^) band from 1376 cm^−1^ to 1337 cm^−1^ between *t* = 5 min and *t* = 38 days is accompanied by an increase in band intensity. This can be explained by an increase in π-electron delocalization and/or formation of more delocalized polaron structures with an increase in reaction time^34^, indicating an increased electrical conductivity in the final products^8^. The shoulder at 1325–1316 cm^−1^ seen in the spectra for t ≥ 5 min can also be associated with *ν* (C–N^•+^) vibrations of the conductive PANI-ES-like form, probably in more delocalized polaron structures^34^. According to previous reports on conducting PANIs (ref. 34 and references cited therein), the coexistence of the two bands in the wavenumber range 1400–1300 cm^−1^ indicates that two differently organized polarons are present in the final product. In our case, two polaronic sites are present, one with relatively lower delocalization–the stronger peak at 1376 –1337 cm^−1^ –and one with highly delocalized polaronic sites–the shoulder at 1325–1316 cm^−1^. This means that the distribution of semiquinone radical cations in poly(PADPA) is not uniform. However, this possibility is not clearly supported by the *in situ* EPR measurements which indicate that one type of radical dominates the final as-formed product (see above, Fig. 3d at *t* = 120 min). It is interesting to note that the Raman spectrum of the final poly(PADPA) vesicle suspension recorded ca. 15 months after the beginning of the synthesis (Supplementary Fig. S3) is very similar to the spectrum recorded at *t* = 38 days (Fig. 4) and shows a very strong band at ≈1330 cm^−1^, indicative for the good conductivity. This demonstrates the remarkably high environmental stability of poly(PADPA) obtained with AOT vesicles as templates.

#### Bands characteristic of benzenoid units

The bands at ≈1230 cm^−1^ and ≈1270 cm^−1^ can be attributed to C–N stretching vibrations in benzenoid (B) units, *ν* (C–N)_B_. The band due to the C–H bending in-plane vibration of the B ring, *δ* (C–H)_B_, is observed at 1182–1186 cm^−1^.

#### Bands characteristic of phenazine- and phenoxazine-type units

In the spectrum recorded after 1 min, a strong band is observed at 1640 cm^−1^ with a similar intensity as the band at 1596 cm^−1^ (C–C vibrations in SQ and Q structures). The band at 1640 cm^−1^ is usually assigned to C~C ring-stretching vibrations in phenazine-, *N*-phenylphenazine- and/or phenoxazine-type units mixed with the C~C stretching vibration of the B ring, *ν* (C~C)_B_ (Fig. 1)^34^,^35^,^36^,^37^. This band is much sharper and stronger in comparison to the broad band observed at ≈1640 cm^−1^ in the spectrum of PADPA/AOT vesicles (Supplementary Fig. S2, spectrum e). It can be seen that the band at 1640 cm^−1^ shifts to lower wavenumbers with an increase of the reaction time (*e.g.*, to 1634, 1630, 1627, and 1625 cm^−1^ at *t* = 5, 15, 20, and 120 min, respectively, Fig. 4). This band shift can be explained by taking into account an increased contribution from the *ν* (C~C)_B_ vibrations (as present in ordinary PANI-like units) and a concomitant decrease in the contribution of C~C ring-stretching vibrations of phenazine-type units with increasing reaction time. In most of the spectra obtained at *t* ≥ 5 min, the intensity of the band at 1640–1621 cm^−1^ is lower than the intensity of the band at 1596 cm^−1^(C–C vibration in SQ and Q structures). This is an indication that the content of ordinary PANI-like Q and SQ segments increases abruptly at *t* ≥ 5 min.

Further bands typical for substituted phenazine- and/or phenoxazine-type of segments^34^,^36^ are also seen in all spectra: at ≈1566 cm^−1^ (weak band) for *t* = 1–120 min, at ≈1572 cm^−1^ for *t* = 1–38 days, and at 1417–1408 cm^−1^ for *t* = 1 min– 3 days. The relative intensities of the bands at 1566 cm^−1^ and 1408–1417 cm^−1^ decrease with an increase of the reaction time, and becomes very weak after *t* = 1 day and *t* = 3 days. An abrupt intensity decrease of “the phenazine band” at 1408–1417 cm^−1^ is observed in the spectrum measured at *t* = 120 min, while in the spectra recorded later on, this band is very weak (*t* = 1 day, 3 days) or absent (*t* = 18 days, 38 days).

#### Unclear assignment

In the spectrum recorded after *t* = 1 day there is a new band located at 1385 cm^−1^ which is not present in the spectra measured at *t* ≤ 120 min. This indicates that there are still changes in the chemical structure of the obtained products beyond *t* = 120 min, which are not clearly reflected in the Uv/vis/NIR spectra: the band intensity at about 1385 cm^−1^ decreases until it is very weak at *t* = 38 days. The appearance of the band at 1385 cm^−1^ after *t* = 1 day occurs simultaneously with the disappearance of “the phenazine band” at ≈1408 cm^−1^. The origin of the band at about 1385 cm^−1^ is unclear at the moment. Possible assignments are *ν* (C–N^+^)/ring stretching vibrations in *N*-phenylphenazine (safranine) and/or phenoxazine type of units^11^,^34^,^37^. Changes in the Raman spectrum of chemically synthesized PANI films during aging are also known^31^, although the aging conditions in that case were treatement at 80 °C for three months.

### Reactions of PADPA with TvL/O_2_ in the Absence of Vesicles (Template-free System) Analysed by *in situ* Raman Spectroscopy Measurements

The changes in the *in situ* Raman spectrum after adding TvL (≈64 nM) to the reaction mixture containing PADPA (1.0 mM) in the *pH* = 3.5 solution without vesicles are shown in Fig. 5. There are clear differences in the outcome of the reaction between this template-free system and the vesicle system.

#### Bands characteristic of quinonoid and semiquinonoid structures

An important spectral difference between the reaction with and without vesicles is the intensity of the ν (C=N)_Q_ band at ≈1498 cm^−1^, which is much higher for the template-free system than for the system with vesicles. In the template free-system it increases with an increase of the reaction time (Fig. 5). This band is typical for the emeraldine base form of PANI, PANI-EB (Fig. 1), and becomes the strongest band in the spectra recorded between *t* = 1 day and *t* = 38 days (Fig. 5). This indicates a much higher content of quinonediimine segments in poly(PADPA) (Fig. 1) in the template-free system than in the vesicle system. Therefore, at least some of the reaction products obtained without vesicles resemble the structural units present in PANI-EB.

In all spectra of the products obtained without vesicles, the intensity of the *δ* (N–H) band at ≈1516 cm^−1^ (indicative of protonation and the presence of SQ rings) is lower than the intensity of the *ν* (C=N)_Q_ band at ≈1497 cm^−1^ (up to *t* = 70 min), while for the spectra of the products obtained in the presence of vesicles (Fig. 4) the opposite is true: the *δ* (N–H) band becomes stronger than the *ν* (C=N)_Q_ band (for *t* > 20 min). In the spectra of the template-free system recorded after *t* = 1 day or *t* = 38 days (Fig. 5), the peak at ≈1516 cm^−1^ is hardly visible.

#### Bands characteristic of polaron structures

One of the most important difference between the *in situ* Raman spectra of the reaction products obtained in the presence of AOT vesicles (Fig. 4) and in the absence of the vesicles (Fig. 5) is the intensity of the *ν* (C–N^•+^) band. In contrast to the spectra of the products obtained in the presence of vesicles−where the *ν* (C–N^•+^) band at ≈1350–1370 cm^−1^ is quite strong already in the early stages of the reaction (*t* = 5–15 min) and its intensity increases with time–the *ν* (C–N^•+^) band (1331–1350 cm^−1^) in the template-free system has a relatively low intensity all the time, even after *t* = 70 min and later at *t* = 1 day or *t* = 38 days (Fig. 5). This is an indication of a much lower content of polaron structures in the products from the template-free system, most probably resulting in a much lower conductivity in comparison to the products obtained in the presence of the vesicles. In addition, in contrast to the system with vesicles, the peak at about 1320 cm^−1^ due to *ν* (C–N^•+^) vibrations, also indicative of highly delocalized polaron structures, is not observed in the spectra of the template-free system. This correlates with the findings from the UV/vis/NIR (no band around 1000 nm) and the EPR measurements (very low signal intensity) for the template-free system.

#### Bands characteristic of phenazine- and phenoxazine- type units

The band attributable to phenazine ring stretching vibrations is observed in the first spectrum (*t* = 1 min) at 1413 cm^−1^ (Fig. 5). This band is significantly weaker in the spectrum recorded after *t* = 5 min, and it becomes very weak, or is even absent, in the spectra measured at longer reaction times. This behaviour is different from the spectra of the products obtained in the presence of the vesicles (Fig. 4), where a band at ≈1413 cm^−1^ is clearly seen in all spectra from *t* = 1 min to *t* = 120 min.

#### Unclear assignment

A new band appears at 1390 cm^−1^ in the spectrum at *t* = 5 min (Fig. 5), and this band is also present (at 1385–1389 cm^−1^) in the spectra taken after longer reaction times. The appearance of a band at this frequency is also observed for the reaction in the presence of the vesicles, see above. The origin of the band is under discussion. As mentioned above, it can be tentatively assigned to the *ν* (C–N^+^)/ring stretching vibrations in *N*-phenylphenazine (safranine) and/or phenoxazine^11^- type of units^34^,^37^. Indeed, pure phenoxazine dyes show a strong Raman band^34^,^38^ at 1390 cm^−1^, and phenosafranine and its polymerization product give Raman bands at 1380 cm^−1^ and 1390 cm^−1^, respectively^37^. After long reaction times (*t* = 38 days), the band is no more present, indicating that chemical changes^31^ may still take place during prolonged storage of the reaction mixture.

#### *Bands indicating C*=*O bonds*

Weak bands at 1665 cm^−1^ and 1684 cm^−1^ observed in the first spectrum (*t* = 1 min) (Fig. 5) can be attributed to stretching vibrations of C=O bonds, indicating that partial hydrolysis of iminoquinonoid C=N bonds took place^11^. This suggestion correlates with previous findings from preliminary ESI-MS measurements that oxygen-containing species are present in reaction intermediates extracted from the template-free system^1^.

### Main Findings from the *in situ* Raman Spectroscopy Measurements

Although there are still uncertainties in the assignment of the bands^33^, the main findings can be summarized as follows: the products formed in the presence of vesicles exhibit (i) a much higher content of polaron structures, (ii) a significantly higher protonation level, and (iii) a much lower content of quinonediimine segments than the products formed without vesicles. The spectra of the system with vesicles indicate a continuous increase in π-electron delocalization with increasing reaction time (which would mean an increase in electrical conductivity). This feature is not observed for the template-free system, where the band characteristic for polaron structures retains a relatively low intensity all the time.

A comparison of the *in situ* Raman spectrum of poly(PADPA) obtained enzymatically with the vesicles with the Raman spectrum of solid, chemically synthesized PANI-ES^39^ shows that there are differences in the molecular structure of the two types of products (Supplementary Fig. S5). On the other hand, the Raman spectrum of poly(PADPA) obtained in the absence of vesicles is remarkably similar to that of chemically synthesized PANI-EB (Supplementary Fig. S6). Furthermore, upon isolation of poly(PADPA) obtained enzymatically in the presence of vesicles, the Raman spectrum changes in comparison to the Raman spectrum recorded *in situ* (Supplementary Fig. S7). This may be due to oxidative intramolecular cyclization of branched units present in the formed products^11^,^34^,^40^,^41^.

### Cyclic Voltammetry Measurements

For the cyclic voltammetry (CV) measurements, the reaction products were deposited onto a glassy carbon (GC) electrode. This was done in two different ways, either (i) by using the isolated and purified poly(PADPA) products deposited in the form of a thin film with additives or (ii) by drop-casting the reaction mixture as obtained (no product isolation). In a first set of measurements, both approaches were applied for the investigation of the redox activity of the poly(PADPA) products obtained in the system with AOT vesicles, while only the latter one–without product isolation–was used to investigate the electrochemical behavior of poly(PADPA) products obtained in the template-free system.

Irrespective of the applied method, the recorded cyclic voltammograms show that poly(PADPA) obtained in the presence of AOT vesicles is redox active up to *pH* 6.00, being the upper limit of the investigated *pH* range (Fig. 6, the first and the second row).

By applying the direct drop-casting method, a complex *pH*-dependent electrochemistry of poly(PADPA) can be observed (Fig. 6, second and third row). At the lowest *pH* of 1.14, the cyclic voltammogram of poly(PADPA) obtained in the presence of AOT vesicles (Fig. 6d) resembles that of PANI, with two anodic peaks observed at 0.27 and 0.62 V *vs*. SCE, and the corresponding cathodic peaks found at 0.18 and 0.42 V *vs*. SCE. Commonly, the couple located at lower potentials is ascribed to the redox transformations of fully reduced chains of the leucoemeraldine form of PANI to the half-oxidized chains of the PANI-ES form and *vice versa*; while the second couple found at higher potentials is due to the interconversion of PANI-ES to the fully oxidized pernigraniline form of PANI^42^,^43^. Upon increasing the *pH* to *pH* = 3.04 a new anodic peak at ~0.4 V (Fig. 6e) was seen in the middle of the cyclic voltammogram, which can be attributed to incorporated phenazine-like structural units^44^. This peak is also seen in the cyclic voltammogram for *pH* = 6.00, but shifted to ~0.25 V (Fig. 6f). The observed shift of all oxidation peaks to less positive potentials with increasing *pH* indicates an easier oxidation process in more alkaline conditions, in accordance with previous findings for aniline monomer and oligomers^40^,^41^ or phenazine derivatives^45^. The observed complex redox behavior as a function of *pH* is caused (i) by reduced levels of protonation of poly(PADPA) upon increasing the *pH* of the supporting electrolyte, (ii) by the presence of segments different from ordinary PANI-like segments (having different *pK*_a_ values), and (iii) by the presence of different anions^8^,^43^, whereby it is difficult to isolate their individual contributions to the observed behavior. This also holds for the *pH*-dependent current peak shift, which is most obvious for the cyclic voltammograms shown in Fig. 6d–f. Although the peak shift towards lower potentials upon increasing the *pH* suggests simultaneous charge transfer and protonation, the complexity of the current response makes it rather difficult to determine the proton-to-electron ratio associated with the corresponding voltammetric peaks observed in the cyclic voltammograms.

Just like the obvious spectroscopic (and clearly visual) differences between the reaction products obtained from PADPA and TvL/O_2_ in the presence of AOT vesicles and the ones obtained without AOT, the electrochemical behavior of the two poly(PADPA) products differs to a great extent. While the cyclic voltammogram of the poly(PADPA)-AOT products at *pH* = 1.14 corresponds well to the cyclic voltammograms of “standard” PANIs, the cyclic voltammogram of poly(PADPA) obtained without vesicles at the same *pH* is quite different, showing two very close oxidation peaks, which almost merged into one. The CV results thus support findings from Raman spectroscopy regarding the positive template effect of AOT vesicles on the formation of conductive PANI-ES-like poly(PADPA) products. The redox activity of poly(PADPA) obtained in the presence of AOT vesicles was preserved even at *pH* = 6.00, (Fig. 6c,f) which is a rather important finding because, usually, PANI loses its electro-activity at *pH* > 5^46^. For poly(PADPA) synthesized in the template-free system, the redox activity was lost already at *pH* = 3.04 (Fig. 6, third row), in contrast to the products obtained with vesicles which retained their redox activity also at *pH* = 6.00. These differences are likely due to the lower protonation level of poly(PADPA) in the template-free system, and also to a much lower conductivity, as compared to poly(PADPA) obtained in the presence of AOT vesicles. This is in agreement with the EPR and Raman spectroscopy measurements which both indicate a higher radical content in poly(PADPA) obtained in the presence of the vesicles, as compared to the reaction products obtained without vesicles, see above. Hence, the CV measurements clearly show that the presence of AOT vesicles is beneficial for the preservation of the redox activity of poly(PADPA) products at high *pH*. This is an important property for possible applications in which electrodes are coated with a conductive organic material^47^,^48^,^49^,^50^,^51^.

## Conclusions

**Figure 2:**
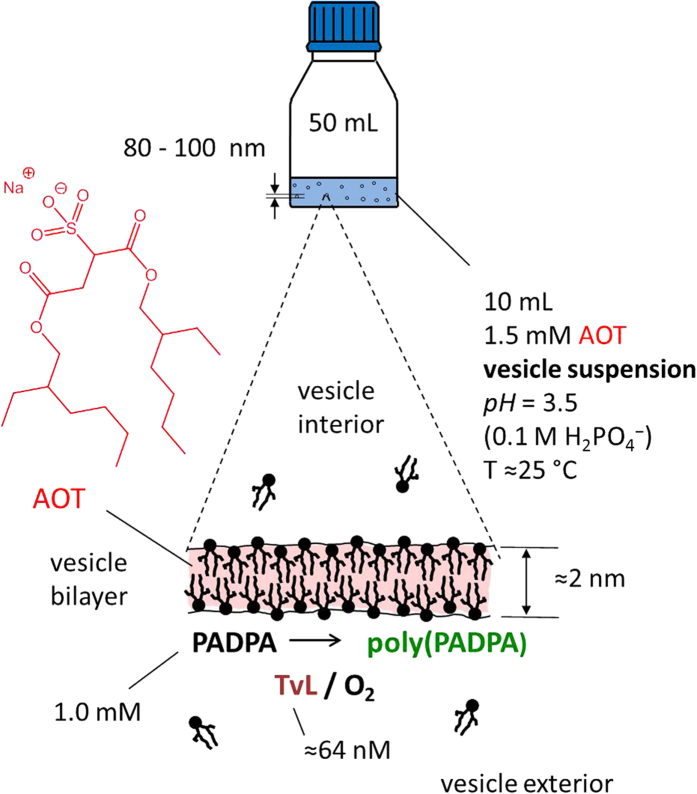
Schematic representation of the reaction setup for the *Trametes versicolor* laccase (TvL)/O_2_-catalyzed oxidation and oligomerization of PADPA to poly(PADPA) in the presence of AOT vesicles as templates. Details of the optimal reaction conditions for obtaining poly(PADPA) which resembles PANI-ES are given, see also Junker *et al*.^1^.

**Figure 3:**
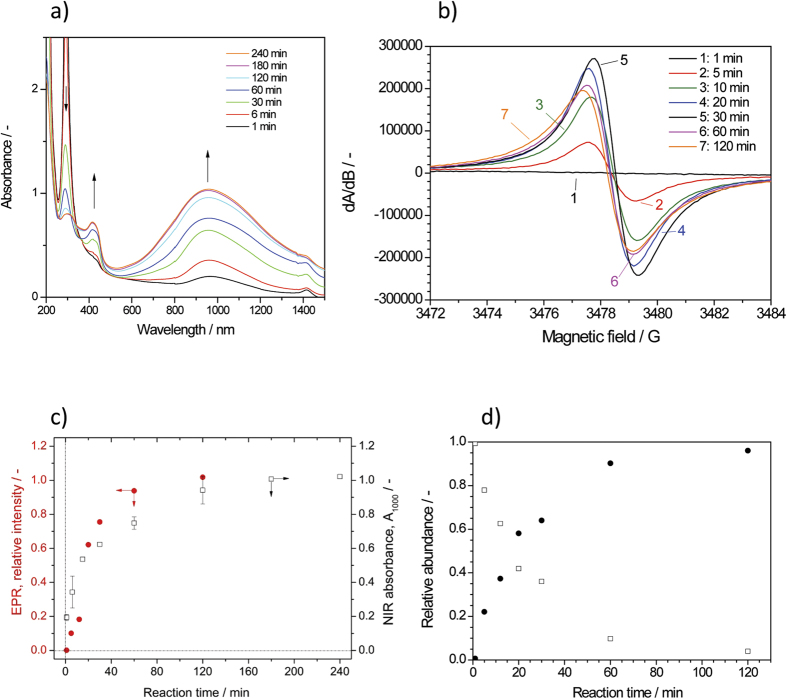
UV/vis/NIR and EPR spectra of the reaction mixture. Changes in the UV/vis/NIR spectrum **(a)** and of the EPR spectrum **(b)** of the reaction mixture during the TvL/O_2_-catalyzed oxidation and oligomerization of PADPA in the presence of AOT vesicles. The reaction times at which the reaction mixtures were analyzed are indicated. (**c**) Changes of the EPR signal integral *vs.* time for the radical species of the reaction product obtained with the vesicles (with estimated errors of ± 3%), overlaid with A_1000_ *vs*. time from the UV/vis/NIR measurements. Note that the intensity of the EPR signal was scaled in order to compare the two signals more easily. For A_1000_, mean values and standard deviations are given (as obtained for 3 or 4 independent reactions). The EPR data are the ones obtained from single measurements. (**d**) The changes of the relative abundance of the two radical species mainly present in the reaction mixture during the reaction (as obtained from fitting of the EPR spectra, see *Methods*) are plotted against the reaction time. Reactions conditions: [AOT] = 1.5 mM, [PADPA]_0_ = 1.0 mM, [TvL] ≈64 nM, pH = 3.5 solution (0.1 M H_2_PO_4_^−^ + H_3_PO_4_), T ≈ 25 °C.

We have shown that *in situ* Raman spectroscopy measurements are very suitable–and complementary to UV/vis/NIR and EPR measurements–for following the *Trametes versicolor* laccase-catalyzed oxidation and oligomerization of PADPA in aqueous media consisting of stable dispersed vesicles. The reaction intermediates and products remain finely dispersed throughout the reaction which allows for easy and highly reproducible measurements without any need for forming films on a solid surface. Due to the complexity of the reaction mixtures investigated, the recorded Raman spectra show complex band patterns, which reflects the various structural units formed during the reaction in the vicinity of the vesicle surface. This complexity makes a complete and unambiguous band assignment difficult, and one has to rely on previous, currently accepted, assignments done on related samples^33^. Nevertheless, all four methods presented here clearly confirm the positive effect the vesicles have–if applied under optimal reaction conditions (Fig. 2)–on the progress and outcome of the reaction. The main conclusions which can be drawn from our investigation are the following:

- There is one main radical type in the poly(PADPA) products obtained in the presence of the vesicles (Fig. 3d) which evolves during the reaction at the expenses of another radical species which dominates at the very early stage of the reaction (most probably PADPA^•+^). Without vesicles, the radical content in the products obtained is much lower^1^ (EPR spectroscopy measurements).
- Bands which are characteristic for PANI-ES are present in the Raman spectrum of poly(PADPA) obtained in the presence of the vesicles with TvL/O_2_, indicating that the main products must contain PANI-ES-like structural units. On the other hand, the Raman spectrum of poly(PADPA) synthesized with TvL/O_2_ in the absence of vesicles is very similar to that of chemically synthesized PANI-EB, indicating low conductivity of such poly(PADPA) products.
- Although poly(PADPA) obtained with TvL/O_2_ in the presence of AOT vesicles is a PANI-ES-like product, several *additional* bands in the Raman spectrum of the final poly(PADPA) product–most likely originating from phenazine and/or phenoxazine-type units–indicate that its molecular structure is somewhat different from (*e.g.*, more complex than) the molecular structure of PANI-ES prepared chemically with APS (ammonium peroxydisulfate). Clearly, “as-obtained” poly(PADPA) is different from PANI-ES prepared chemically with APS (Supplementary Fig. S5, Raman spectroscopy measurements).
- During the isolation of poly(PADPA) from the poly(PADPA)-AOT vesicle suspension, chemical reactions seem to take place which lead to modifications of the product(s). This is important to keep in mind if the physical properties of solid, isolated poly(PADPA)-AOT products^52^,^53^ are compared with the physical properties of “as-obtained” products.
- The “as-obtained” poly(PADPA)-vesicle suspension can be used directly for coating glassy carbon electrodes (drop-casting), whereby the adsorbed poly(PADPA) products are redox active, as expected for PANI-ES-like products (CV measurements). At *pH* = 1.14, the cyclic voltammogram of poly(PADPA)-AOT products is very similar to the cyclic voltammograms of conductive ‘standard’ PANIs, thus supporting the results of Raman spectroscopy regarding the positive template effect of AOT vesicles on the formation of conductive PANI-ES-like poly(PADPA) products. In addition, the redox activity of poly(PADPA) produced with vesicles is preserved at higher *pH* values, up to the upper limit of the investigated *pH* range of *pH* = 6.00, unlike conventional PANI-ES which completely loses its redox activity at *pH* > 5^46^ or even 4^54^ and unlike poly(PADPA) produced without vesicles which loses its redox activity already at *pH* = 3.0.

A final challenge is to determine more precisely the chemical structure of poly(PADPA). This includes the molar masses, a final proof of the expected *para*-coupling of the PADPA repeating units–as they are expected to be present in PANI-ES–and a verification/disproof of the presence of phenazines and/or phenoxazine –type units, as suggested from the Raman spectroscopy analysis (see above). Work towards reaching this goal by using a detailed HPLC analysis in combination with mass spectrometry is in progress. More from an application point of view, the suitability of the direct use of the “as obtained” poly(PADPA)/AOT vesicle suspension for bioelectrode fabrication needs to be investigated. At least with respect to the first CV results obtained in this work, it seems appropriate to continue research in this field, and to compare the advantages and disadvantages of the PANI-ES-like poly(PADPA)–as obtained from PADPA in the present work with TvL/O_2_ and AOT vesicles–with, for example, PANI-ES as obtained from aniline with horseradish peroxidase isoenzyme C/H_2_O_2_ and the same type of vesicles^55^, even though we know that in this latter case much more enzyme is required for the synthesis than in the case of TvL/O_2_ and PADPA^1^.

The Raman spectroscopy analysis we present here is the first one for a vesicle-assisted enzymatic polymerization reaction. There is no doubt that the type of *in situ* Raman spectroscopy measurements shown here can also be used in the future for investigating other similar enzymatic polymerizations with vesicles as structure-directing agent. This will allow a direct comparison among related enzymatic systems.

## Methods

### Chemicals

Laccase from *Trametes versicolor* (TvL, EC 1.10.3.2; product no. 51639, 13.6 U mg^−1^, lot no. BCBF7247 V), sodium bis(2-ethylhexyl) sulfosuccinate (AOT ≥ 99%), sodium phosphate monobasic (NaH_2_PO_4_), chloroform (ReagantPlus ≥ 99.8%, 0.5-1.0% ethanol as stabilizer), 2,2-Diphenyl-1-picrylhydrazyl (DPPH, 95%), and *N*-phenyl-1,4-phenylendiamine (=*p*-aminodiphenylamine, PADPA, 98%) were purchased from Sigma-Aldrich. PADPA was purified by multiple recrystallizations from hexane until white crystals were isolated. Analytical grade ethanol was purchased from Scharlau. Phosporic acid (H_3_PO_4_, 85%) and Methyl-*tert*-butyl ether (MTBE, ≥99.0%) were bought from Fluka. All aqueous solutions were prepared with Milli-Q water.

### The *pH* = 3.5 Solution

A phosphate solution of *pH* = 3.5 was made by weighing the appropriate amount of NaH_2_PO_4_, dissolving it in Milli-Q water for obtaining a 0.1 M solution, and adjusting the *pH* to *pH* = 3.5 by adding the necessary amount of a 1 M H_3_PO_4_ solution. This *pH* = 3.5 solution mainly consists of dihydrogenphosphate (H_2_PO_4_^−^) with a total H_2_PO_4_^−^ + H_3_PO_4_ concentration of about 0.1 M; it will hereon be referred to as ‘*pH* = 3.5 solution’.

### PADPA Stock Solutions

Two different PADPA stock solutions were prepared, one (1.5 mM) by using the *pH* = 3.5 solution, the other (150 mM) by using ethanol. For the *in situ* Raman and UV/vis/NIR spectroscopy measurements, the 1.5 mM PADPA solution was used (always freshly prepared for the day of use). In order to dissolve the PADPA in the *pH* = 3.5 solution, the required amount of purified PADPA was first dispersed in the *pH* = 3.5 solution, and the resulting suspension was shaken vigorously for ca. 30 min, placed in an ultrasound bath (Bandelin Sonorex RK 100 H) for 15 min, and subsequently shaken vigorously for another 15 min. The *pH* value was then adjusted to *pH* = 3.5 with the necessary amount of a 1 M H_3_PO_4_ solution. Reactions carried out by using this aqueous 1.5 mM PADPA stock solution are referred to as *conventional method* (see below). For the EPR measurements, a stock solution of PADPA (150 mM) in ethanol was prepared, of which the necessary amount was directly added to the reaction mixture (*injection method*).

### TvL Stock Solution

A TvL stock solution was prepared by dissolving 12.92 mg of the commercial laccase product in 1 ml of water by vortexing, followed by 2 min of centrifugation at 16,000 rpm (Eppendorf centrifuge 5415 D) and subsequent removal of the supernatant. With the analytical method described previously^6^, the concentration of TvL in the stock solution (the supernatant) was found to be ≈16 μM. This solution can be stored in the refrigerator at *T *≈ 4 °C for up to 1 month without significant loss in laccase activity.

### AOT Vesicles Preparation

Large unilamellar vesicles were prepared by polycarbonate membrane extrusion^1^. A weighted sample of 0.178 g AOT was dissolved in a small amount (typically ca. 10 ml) of chloroform in a 250 ml glass round-bottomed flask. The chloroform was slowly removed on a rotary evaporator, thus producing a thin film of the AOT on the glass surface. The film was dried on high vacuum overnight and subsequently suspended in 20 ml of the *pH* = 3.5 solution to form the AOT stock solution with [AOT] = 20 mM. The resulting suspension underwent 10 freeze-thaw cycles in liquid nitrogen and a 60 °C water bath. Finally, the suspension was extruded 5 times through a Nucleopore^®^ polycarbonate membrane with a 200 nm pore size and 10 times through a polycarbonate membrane with a 100 nm pore size. The vesicles were subsequently characterized by dynamic light scattering (DLS) and were determined to have an average diameter of about 80–100 nm^56^. They were stored at room temperature and protected from light and used within one month after preparation.

### Reaction of PADPA with TvL/O_2_ in the Presence and Absence of AOT Vesicles

The reaction of PADPA with TvL/O_2_ was always conducted by first adding into a reaction flask the *pH* = 3.5 solution, then the AOT vesicle suspension (20 mM), then the PADPA stock solution (1.5 mM in the *pH* = 3.5 solution or 150 mM in ethanol), and finally the aqueous TvL stock solution (≈16 μM) to initiate the reaction. For the template-free reaction without vesicles, the vesicle stock suspension was replaced with the corresponding volume of the *pH* = 3.5 solution. We found that the kinetics of the reaction may depend on the type and geometry of the reaction vessel and the dimension of the surface area of the reaction volume which is in contact with air. Therefore, all reactions, which were analyzed by *in situ* measurements using UV/vis/NIR, EPR and Raman spectroscopy were carried out in the same way. Typically, a 50 mL Schott Duran^®^ laboratory glass bottle was used with a reaction volume, V_rxn_, of 10 mL. During the reaction, the bottle was kept closed with a polypropylene screw cap in order to avoid evaporation of water and the concomitant concentration changes. The reaction was carried out in two different ways, either with the method we used previously (*conventional method*), or with the *injection method*. Both methods yielded the same UV/vis/NIR spectra. For the 10 mL reaction volume with the *conventional method*, 2.56 mL of the *pH* = 3.5 solution was added to the bottle, then 0.75 mL of the AOT vesicles suspension, then 6.67 mL of the 1.5 mM PADPA stock solution (*pH* = 3.5), and finally 40 μL of the TvL stock solution. For the *injection method* with the same V_rxn_ = 10 mL, 9.17 mL of the *pH* = 3.5 solution was added, then 0.75 mL of the AOT vesicle suspension, then 67 μL of the 150 mM PADPA stock solution (ethanol), and finally 40 μL of the TvL stock solution. The final concentrations were as follows: [AOT] = 1.5 mM, [PADPA] = 1.0 mM, [TvL] = 64 nM, no ethanol (*conventional method*), or 0.67 vol % ethanol (*injection method*). The reactions were run without any stirring.

### *In situ* UV/vis/NIR Spectroscopy Measurements

UV/vis/NIR spectroscopy measurements were conducted on a Jasco-V670 spectrophotometer. Quartz cuvettes with a 1 mm path length (Helma Analytics) were used for all measurements. The background suspension consisted of the AOT vesicles ([AOT] = 1.5 mM) in the *pH* = 3.5 solution. After initiation of the reaction with TvL (*conventional method*), 300 μL samples of the reaction mixture were removed from the reaction mixture at predetermined times, measured, and then discarded.

### *In situ* EPR Spectroscopy Measurements

The EPR spectra were recorded with a Bruker EMX X-band spectrometer (Bruker BioSpin, Rheinstetten, Germany) at room temperature, equipped with a cylindrical TM (transverse magnetic) cavity. Ca. 1 mL (with a Pasteur pipette) of the reaction mixture (V_rxn_ = 10 mL, *injection method*) was transferred into a EPR flat cell (Wilmad Labglass, Vineland NJ, USA) immediately after starting the reaction, *i.e.* after adding TvL, as well as after predetermined time (up to 2 h). For the measurements without vesicles, a small DPPH crystal (<1 mm in diameter) was attached to the flat cell with a scotch tape at the position of the measurement window. Since the *g*-value of DPPH is known with great precision (*g* = 2.0036 ± 0.0001)^57^, the EPR signal of DPPH was used for calibrating the determination of the *g*-value of the signals of radical centers present in poly(PADPA) and in reaction intermediates. The spectra were measured at ≈9.7 GHz with a modulation frequency of 100 kHz and modulation amplitudes of 1 G. The spectra recorded in the presence of the vesicles were fitted by using the software Easyspin^58^, assuming that the measured spectra are composed of only two major individual spectra originating from two different radical species. Please note that in our previous EPR measurements, the reactions analyzed were run in 5 mL polypropylene Eppendorf tubes (*V*_rxn_ = 1 mL), see Junker *et al*.^1^. The general behavior with respect to the outcome of the reaction was the same if compared to the reaction carried out in the way used for the present work (50 mL Schott Duran^®^ laboratory glass bottle, *V*_rxn_, of 10 mL). There are, however, differences between the two systems if the reaction kinetics is compared. Therefore, the EPR measurements presented in this work can not be compared directly with the EPR data presented previously^1^.

### *In situ* Raman Spectroscopy Measurements

**Figure 4:**
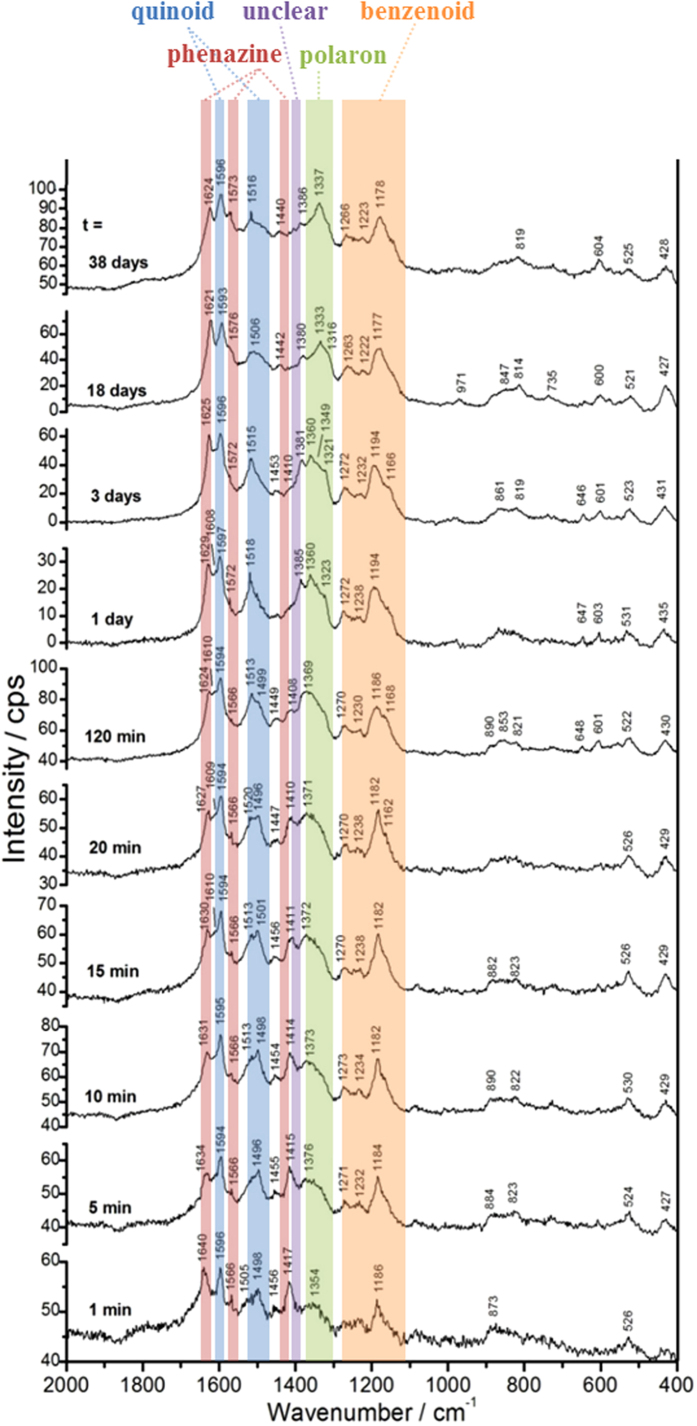
Changes in the Raman spectrum of the reaction mixture during the oxidation of PADPA with TvL/O_2_ in the *presence* of AOT vesicles (*in situ* Raman measurements; each spectrum was recorded for a new aliquot taken from the reaction mixture at specified reaction time and transferred into a sample well at the gold sample support; automatic fluorescence correction was performed for the spectra recorded at 1–38 days; for more details on the Raman measurements see *Methods*, part “*In situ* Raman Spectroscopy Measurements”). For the reaction conditions, see the legend of Fig. 3. The reaction times *t* at which samples were withdrawn from the reaction mixture and analyzed are written near the corresponding spectrum. Excitation wavelength: 633 nm.

**Figure 5:**
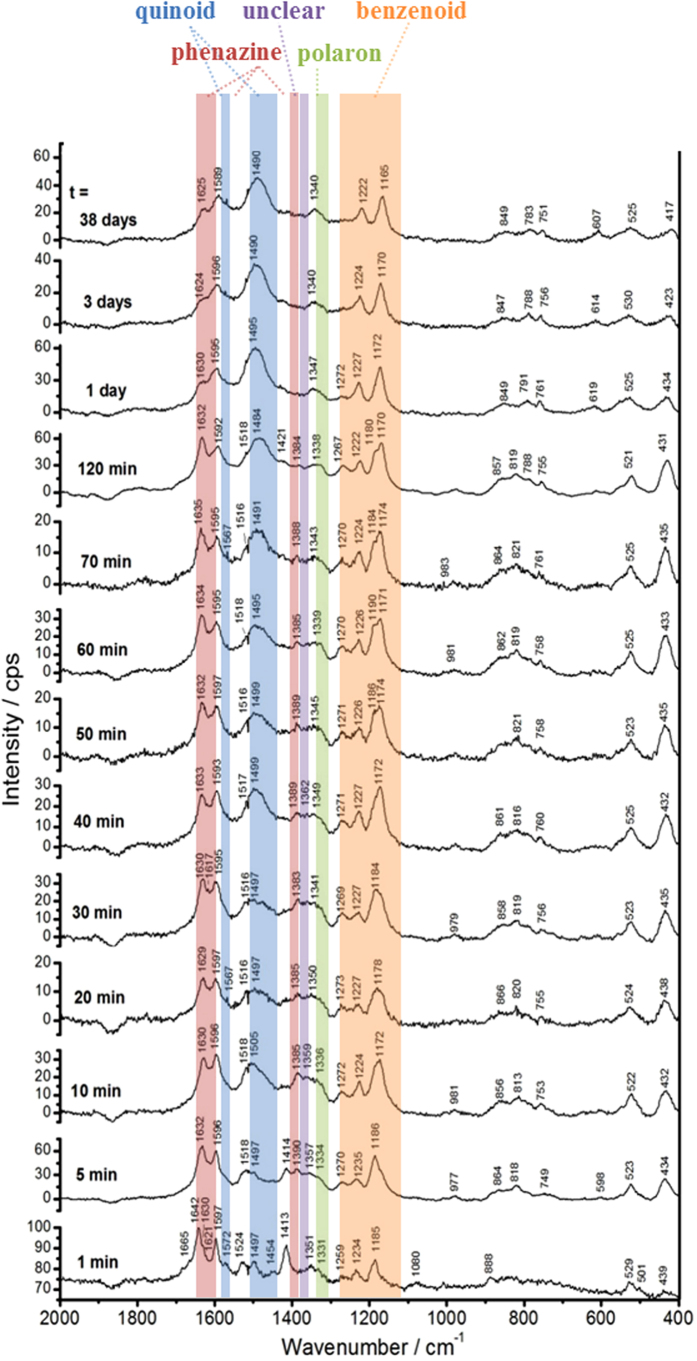
Changes in the Raman spectrum of the reaction mixture during the oxidation of PADPA with TvL/O_2_ in the *absence* of vesicles (*in situ* Raman measurements; each spectrum was recorded for a new aliquot taken from the reaction mixture at specified reaction time and transferred into a sample well at the gold sample support; all spectra, with the exception of that recorded at time 1 min, were obtained after automatic fluorescence correction; for more details on Raman measurements see *Methods*, section “*In situ* Raman Spectroscopy Measurements”). For the reaction conditions, see the legend of Fig. 3. The reaction times *t* at which samples were withdrawn from the reaction mixture and analyzed are written near the corresponding spectrum. Excitation wavelength: 633 nm.

**Figure 6:**
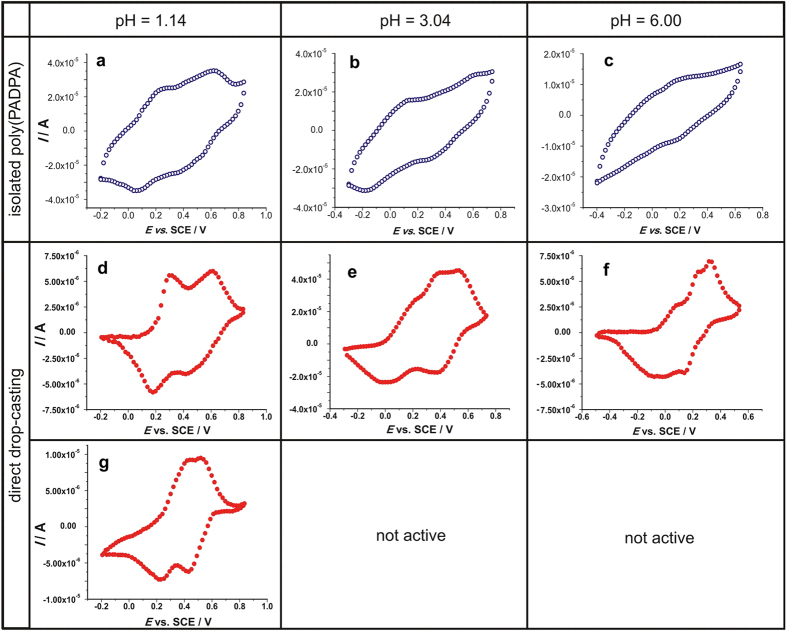
Cyclic voltammograms of GC electrode which was modified either (i) with isolated and purified poly(PADPA) synthesized in the presence of AOT-vesicles (first row‒ (a,b,c), or (ii) by direct drop-casting of the AOT-containing PADPA polymerization system (second row‒(d,e,f) and the PADPA polymerization system without AOT vesicles (third row‒(g). For the experimental conditions for the synthesis of poly(PADPA), see the legend of Fig. 3. Voltammograms were recorded in quiescent N_2_-purged solutions of different *pH* values (first column *pH* = 1.14, second column *pH* = 3.04, and third column *pH* = 6.00) at a common polarization rate of 20 mV s^−1^.

The Raman spectra were recorded with a DXR Raman microscope (Thermo Scientific, Waltham MA, USA), equipped with a research optical microscope and a CCD detector. A HeNe gas laser with an excitation wavelength of 633 nm was used for all measurements. The *in situ* Raman spectra of the reaction products were recorded by withdrawing 5 μL aliquots of the reaction mixtures (prepared with the *conventional method*) from the reaction vessel at specified reaction times and transferring them into sample wells at the sample platform (Gold EZ-Spot Micro Mount sample slide, Thermo Scientific), both for reactions run in the presence and in the absence of AOT vesicles. Each spectrum was measured for a new “drop” of the reaction mixture taken from the reaction vessel (‘macroreactor’) and transferred into the empty and clean sample well (‘microreactor’), Supplementary Fig. S8. Thus, since there was no interrupting of the polymerization reaction for the purpose of Raman measurements, *i.e.,* the spectra of the reaction mixture were recorded during the reaction without isolation of reaction products and changing reaction conditions, these measurements are considered as *in situ* Raman measurements. After filling the well with the sample of the reaction mixture, the slide with the sample was placed on an X-Y motorized sample stage and the laser beam was focused on the sample at an objective magnification of 10×. The scattered light was analyzed by the spectrograph with a 600 lines mm^−1^ grating. The laser power on the sample was kept at 5.0 mW for the spectra of the various reaction components measured before the reaction (Supplementary Fig. S2). For the spectra of the samples of the reaction mixture recorded during the reaction with vesicles (Fig. 4) the laser power was 4.0 mW, and for the spectra recorded during the reaction without vesicles (Fig. 5), the laser power was 2.0 mW and 4.0 mW for reaction times before and after 120 min, respectively. The spectra were recorded about 30 s after transferring the sample drop from the reaction flask into the sample wells. The exposure time was 10 s and 10 exposures per spectrum were applied. In the cases with high fluorescence background, automatic fluorescence correction was performed using the OMNIC software.

### Poly(PADPA) Product Isolation

After 24 h reaction time with V_rxn_ = 30 ml in a 100 ml Schott Duran^®^ laboratory glass bottle (*injection method*), the final products were isolated by extracting them from the reaction mixture with methyl-*tert*-butylether (MTBE). Multiple extraction steps were needed. MTBE was removed *in vacuo*, followed by treating the dried products with 1 M HCl to ensure protonation. Excess HCl was removed, and the products were washed with water by repeated centrifugation and removal of supernatant.

### Raman Spectroscopy Measurements of Isolated Poly(PADPA)

Raman spectra of solid poly(PADPA) products, isolated from the reaction mixtures and purified, were also recorded. These powdered samples were placed on an X-Y motorized sample stage and the laser beam was focused on the sample at an objective magnification ×50. The excitation wavelength and the spectrograph grating were the same as for the *in situ* Raman measurements, but the laser power on the sample was 0.2–0.5 mW.

### Cyclic Voltammetry Measurements

The redox activity of the poly(PADPA) products was probed by using cyclic voltammetry (CV) in two different ways. First, the redox activity of *isolated poly*(*PADPA*) was measured upon modification of a glassy carbon (GC) disk electrode with a solid film of isolated product. The modification was done as follows: 2.5 mg of poly(PADPA) and 1.1 mg Vulcan XC-72 R (conductive carbon black from Cabot, USA) were dispersed in 500 μL of a water/ethanol mixture (3:2 v/v) with the addition of 20 μL of a 5 wt.% Nafion^®^ solution in ethanol and homogenized in an ultrasonic bath. Vulcan XC-72 R was added as a current collector and to increase the conductivity of the poly(PADPA) layer as done previously^59^. After homogenization, 10 μL of the poly(PADPA) suspension was loaded onto a GC disk (Pine, USA; 5 mm in diameter) and dried in N_2_ gas flow. Such a prepared electrode was transferred into an electrochemical cell and subjected to potential cycling. In this way, a total mass of 48 μg of poly(PADPA) product was loaded onto the working GC electrode.

In the second set of the experiments, the redox activity of the poly(PADPA) product was probed by *direct drop-casting of the reaction mixture* on the working GC electrode *without* isolation of the reaction product and *without* the addition of Vulcan XC-72R or any other electronically conductive component. Upon completion of the enzymatic polymerization (5 days), 15 μL of the reaction mixture was drop-casted on the surface of the GC disk electrode and dried under N_2_ gas flow. Such a prepared electrode was transferred into the electrochemical cell and subjected to potential cycling. In this way, a total mass of 2.76 μg of PADPA units (referred to the initial concentration in the reaction mixture) was loaded on the working GC electrode.

CV measurements were done in a conventional one-compartment glass electrochemical cell with a saturated calomel electrode (SCE) and a large Pt foil as reference and counter electrodes, respectively. Measurements were performed using the Gamry PCI4-750 potentiostat (Gamry, USA) at room temperature. The CV measurements were always started from cathodic potentials which depended on the *pH* of the electrolyte solution. 0.1 M HCl (*pH* = 1.14), 1 mM HCl + 0.1 M KCl (*pH* = 3.04) and 0.1 M sodium phosphate buffer (*pH* = 6.00) were used as supporting electrolytes. Prior to the deposition of the poly(PADPA) product on the GC electrode, the disk was polished to a mirror finish using a diamond paste, followed by thorough washing with acetone and deionized water.

## Additional Information

**How to cite this article**: Ležaić, A. J. *et al*. Insight into the template effect of vesicles on the laccase-catalyzed oligomerization of *N*-phenyl-1,4-phenylenediamine from Raman spectroscopy and cyclic voltammetry measurements. *Sci. Rep.* **6**, 30724; doi: 10.1038/srep30724 (2016).

## Supplementary Material

Supplementary Information
