# Erratum: Insight into the template effect of vesicles on the laccase-catalyzed oligomerization of *N*-phenyl-1,4-phenylenediamine from Raman spectroscopy and cyclic voltammetry measurements

**DOI:** 10.1038/srep33770

**Published:** 2016-09-30

**Authors:** Aleksandra Janoševic Ležaić, Sandra Luginbühl, Danica Bajuk-Bogdanović, Igor Pašti, Reinhard Kissner, Boris Rakvin, Peter Walde, Gordana Ćirić-Marjanović

Scientific Reports 6: Article number: 3072410.1038/srep30724; published online: 08 26 2016; updated: 10 07 2016

In the original version of this Article, Peter Walde was incorrectly affiliated with:

University of Belgrade-Faculty of Pharmacy, Department of Physical Chemistry and Instrumental Methods, Vojvode Stepe 450, 11221 Belgrade, Serbia.

The correct affiliation is listed below:

Department of Materials, ETH Zürich, Vladimir-Prelog-Weg 5, CH-8093 Zürich, Switzerland.

This error has now been fixed in the HTML and PDF versions of this Article.

