# Supplementary material for: Insight into the template effect of vesicles on the laccase-catalyzed oligomerization of N-phenyl-1,4-phenylenediamine from Raman spectroscopy and cyclic voltammetry measurements
Source: Sci Rep. 2016 Aug 26;6:30724. doi: 10.1038/srep30724 (PMC4999881; doi:10.1038/srep30724)
Supplement: Supplementary Information [file srep30724-s1.pdf]

Supplementary Information

Insight into the template effect of vesicles on the laccase-catalyzed oligomerization of *N*-phenyl-1,4-phenylenediamine from Raman spectroscopy and cyclic voltammetry measurements

Aleksandra Janoševic Ležaić,[1] Sandra Luginbühl,[2] Danica Bajuk-Bogdanović,[3] Igor Pašti,[3] Reinhard Kissner,[4] Boris Rakvin,[5] Peter Walde,[2]* Gordana Ćirić-Marjanović[3,]*

[1] Faculty of Pharmacy, Department of Physical Chemistry and Instrumental Methods, University of Belgrade, Vojvode Stepe 450, 11221 Belgrade, Serbia

[2] Department of Materials, ETH Zürich, Vladimir-Prelog-Weg 5, CH-8093 Zürich, Switzerland

[3] Faculty of Physical Chemistry, University of Belgrade, Studentski trg 12-16, 11158 Belgrade, Serbia

[4] Department of Chemistry and Applied Biosciences, ETH Zürich, Vladimir-Prelog-Weg 2, CH-8093 Zürich, Switzerland

[5] Division of Physical Chemistry, Institute Ruđer Bošković, Bijenička cesta 54, HR-10000 Zagreb, Croatia

Content

1. UV/vis/NIR and EPR Spectra of the Template-Free Reaction Mixture

2. Reference Raman Spectra of the Solutions Used Before Starting the Reaction

3. *In situ* Raman Spectra of the Poly(PADPA)-AOT Suspension and of the NaH_2PO_4 Solution Measured by Using Different Sample Supports

4. Comparison of the Raman Spectra of Enzymatically Obtained Poly(PADPA) With the Raman Spectra of Chemically Synthesised PANI-ES and PANI-EB

5. Raman Spectroscopy Measurements of Poly(PADPA) Isolated from the Reaction Mixtures

6. Comment on the Sample Preparation for the Cyclic Voltammetry Measurements

7. Set-up for the *In situ* Raman Monitoring

8. References

1. UV/vis/NIR and EPR Spectra of the Template-Free Reaction Mixture

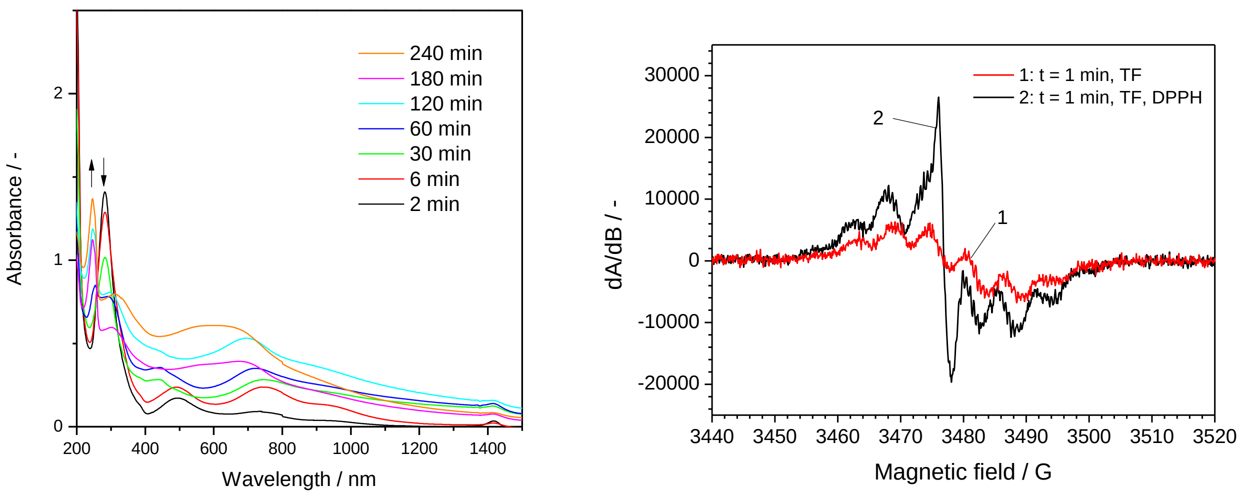

A B

Supplementary Figure S1. A: Changes in the UV/vis/NIR spectrum of the reaction mixture during the TvL/O_2-catalyzed oxidation and oligomerization of PADPA in the absence of vesicles (template-free system). $[PADPA]_0$ = 1.0 mM, [TvL] ≈ 32 nM, pH = 3.5 solution (0.1 M $H_2PO_4^-$ + H_3PO_4), T ≈ 25 °C. The reaction times at which the reaction mixtures were analyzed are indicated; path length l = 0.1 cm. Product precipitation is observed after about 2-3 hours[S1]. Therefore, UV/vis/NIR measurements after this time are no longer meaningful; however, UV/vis/NIR measurements of the template-free reaction system after shorter reaction times are. At the initial stage of the reaction without vesicles, after 2 min, a band at 500 nm is detected. As the reaction proceeds, an additional band at ca. 780 nm starts to develop. After 2 h, the band at 500 nm fades. At reaction times of 3 h and 4 h (when precipitation begins), a 'plateau' from 400 to 700 nm develops because of light scattering. During the entire course of the reaction, one can observe a steady decrease of the band at ≈285 nm (originating from PADPA) and a steady increase of that at ≈250 nm. **B:** EPR spectrum of the reaction product obtained in the absence of vesicles (template free system) after *t* = 1 min, recorded without any reference substance (curve 1), or in the presence of DPPH (curve 2), see *Methods*. The *g*-value for DPPH is 2.0036 ± 0.0001[S2].

2. Reference Raman Spectra of the Solutions Before Starting the Reaction

Before analyzing the changes in the *in situ* Raman spectrum of the reaction mixture as a function of time, reference Raman spectra of deionized water, the *pH* = 3.5 solution, the AOT vesicle suspension (1.5 mM AOT), the PADPA solution (1.0 mM), and the AOT vesicles suspension containing PADPA were recorded (**Supplementary Fig. S2**). The spectrum of deionized water (**Supplementary Fig. S2**, spectrum a) has a broad band at 1637 cm^{-1} due to H–O–H deformation vibrations, δ(O−H)[S3,S4]. This band is also present in the Raman spectrum of the *pH* = 3.5 solution at ≈ 1630 cm^{-1}, together with two additional bands at 1077 cm^{-1} and 879 cm^{-1} (**Supplementary Fig. S2**, spectrum b) which originate predominately from $H_2PO_4^-$(symmetrical stretching vibrations of PO_2 and $P(OH)_2$, respectively)[S5]. The spectrum of the vesicle suspension (**Supplementary Fig. S2**, spectrum c) has two additional bands which are due to AOT: one at 1047 cm^{-1} (from symmetrical stretching vibration of the sulfonate group ($R{-}SO_3^-$)[S6,S7] and from symmetrical C–C bond vibration[S6]), and a second at 1450 cm^{-1} (from CH_2 deformation vibration)[S7]. Both of these bands are also present in the Raman spectrum of neat AOT, at 1070 cm^{-1} and 1461 cm^{-1}; they are the strongest bands between 500 and 2000 cm^{-1} (spectrum not shown). The origin of the band (shoulder) at 1670 cm^{-1} is not clear. It may be due to interactions between water molecules and the AOT vesicle surface. Other smaller bands in the Raman spectrum of neat AOT are too weak to be seen in the spectrum of the vesicle suspension. The Raman spectrum of PADPA (1.0 mM) in the *pH* = 3.5 solution (**Fig. 4**, spectrum d) shows weak bands attributed to PADPA at wavenumbers ≈1600, 1524, 1440 and 1415 cm^{-1} (aromatic ring CC stretching vibrations[S4]), 1345 and 1234 cm^{-1} (C–N stretching vibrations[S4]), and at 1167 cm^{-1} (C–H in-plane bending[S4]), besides the stronger bands of phosphate buffer and water. For the mixture containing PADPA and AOT vesicles in the *pH* = 3.5 solution (**Fig. 4**, spectrum e), the bands of PADPA can be seen at ≈1600, 1415, 1331, and 1242 cm^{-1} (the band of PADPA at 1440 cm^{-1} is overlapped by the band of AOT at 1451 cm^{-1} while the band at ≈1524 cm^{-1} is probably masked by the spike).

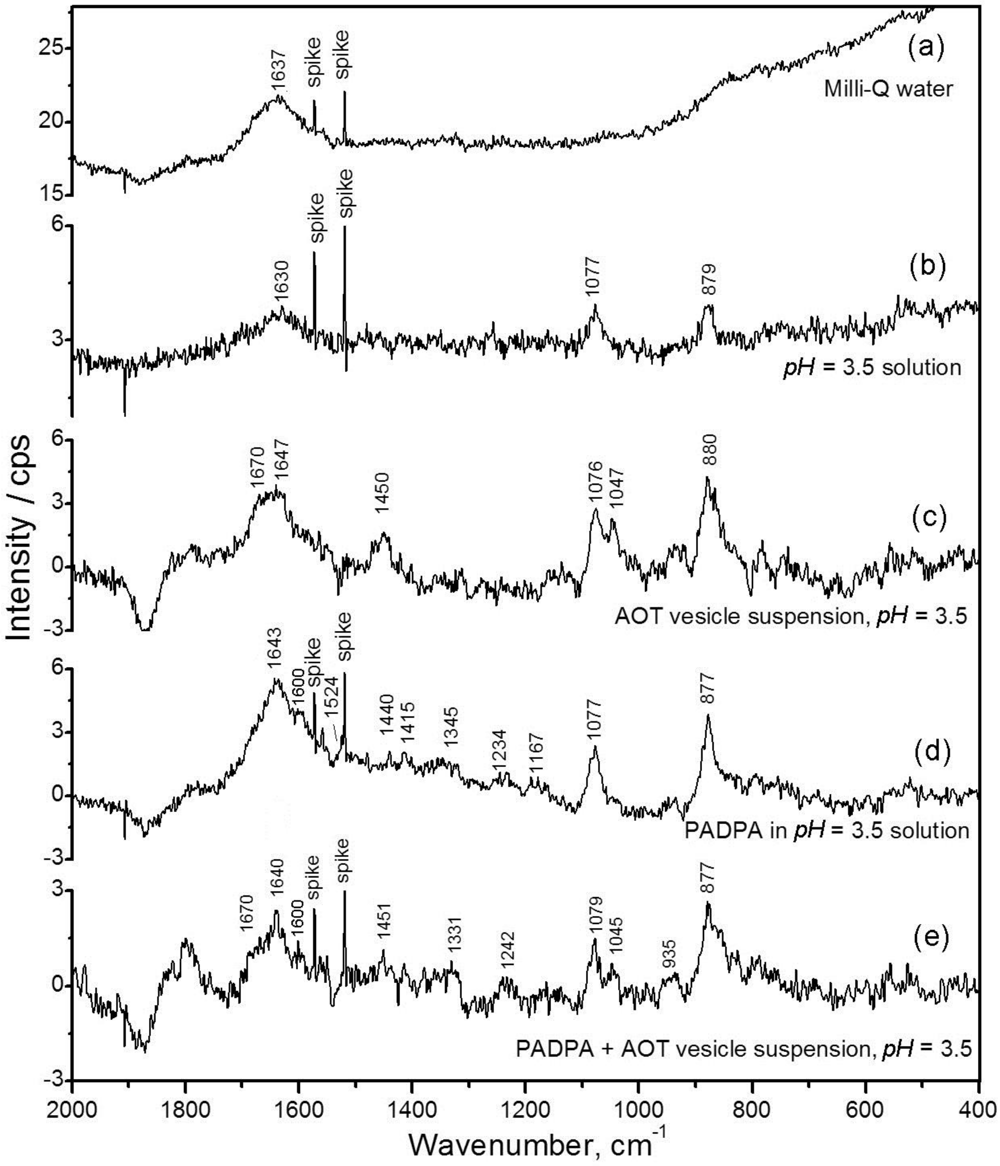

Supplementary Figure S2. Raman spectra of the solutions of the various reaction components measured using gold support (Gold EZ-Spot Micro Mount slide), before the reaction: (a) the Milli-Q water used; (b) the *pH* = 3.5 solution (0.1 M $H_2PO_4^-$ + H_3PO_4); (c) the AOT vesicle suspension (1.5 mM AOT, *pH* = 3.5); (d) the PADPA solution (1.0 mM dissolved in the *pH* = 3.5 solution); (e) the PADPA/vesicle suspension (1.0 mM, 1.5 mM AOT, *pH* = 3.5 solution). Excitation wavelength: 633 nm. All spectra, with the exception of (a), were obtained after automatic fluorescence correction. For more details on Raman measurements see *Methods*, section "*In situ* Raman Spectroscopy Measurements". Please note that the sharp peaks at ≈1518 and ≈1572 cm^{-1} are instrumental artifacts and are seen with variable intensity in all spectra. They are marked as "spike".

3. ***In situ*** **Raman Spectra of the Poly(PADPA)-AOT Suspension and of the NaH_2PO_4 Solution Measured by Using Different Sample Supports**

In situ Raman spectra of the final poly(PADPA) suspension (produced enzymatically by TvL/O_2 in the presence of AOT vesicles) were recorded using different sample supports (**Supplementary Fig. S3**) to check the potential influence of the type of support on the spectra. As can be seen, the Raman spectra obtained using Al foil (**Supplementary Fig. S3 d**), CaF_2 microscope slide (**Supplementary Fig. S3 b**) and Gold EZ-Spot Micro Mount slide (**Supplementary Fig. S3 c**) as a support are almost identical - there is no shift or enhancement of certain bands in the case of the chosen Gold EZ-Spot Micro Mount sample slide. This confirms that there are no interactions between the Gold EZ-Spot Micro Mount slide as a support used for the *in situ* Raman measurements in the present work and the reaction mixture sample, *i.e.* the influence of the sample support on the spectra is negligible in our measurements. The spectrum acquired using ordinary glass microscope slide (**Supplementary Fig. S3 a**) consists predominately of strong fluorescence background (well known feature in the literature) which masks the Raman bands of the sample. This is the reason why ordinary microscope glass slides can not be used as a support for our liquid suspension samples and 633 nm excitation. The absence of interactions between the used support (Gold EZ-Spot Micro Mount slide) and the *transparent* liquid sample is also justified by the Raman measurements of the aqueous solution of 0.1 M NaH_2PO_4 using different sample supports (**Supplementary Fig. S4**). Again, it can be seen that the Raman spectra obtained using Al foil (**Supplementary Fig. S4 d**), CaF_2 microscope slide (**Supplementary Fig. S4 b**) and Gold EZ-Spot Micro Mount slide (**Supplementary Fig. S4 c**) are very similar. High fluorescence background is again observed when an ordinary glass microscope slide is used (**Supplementary Fig. S4 a**).

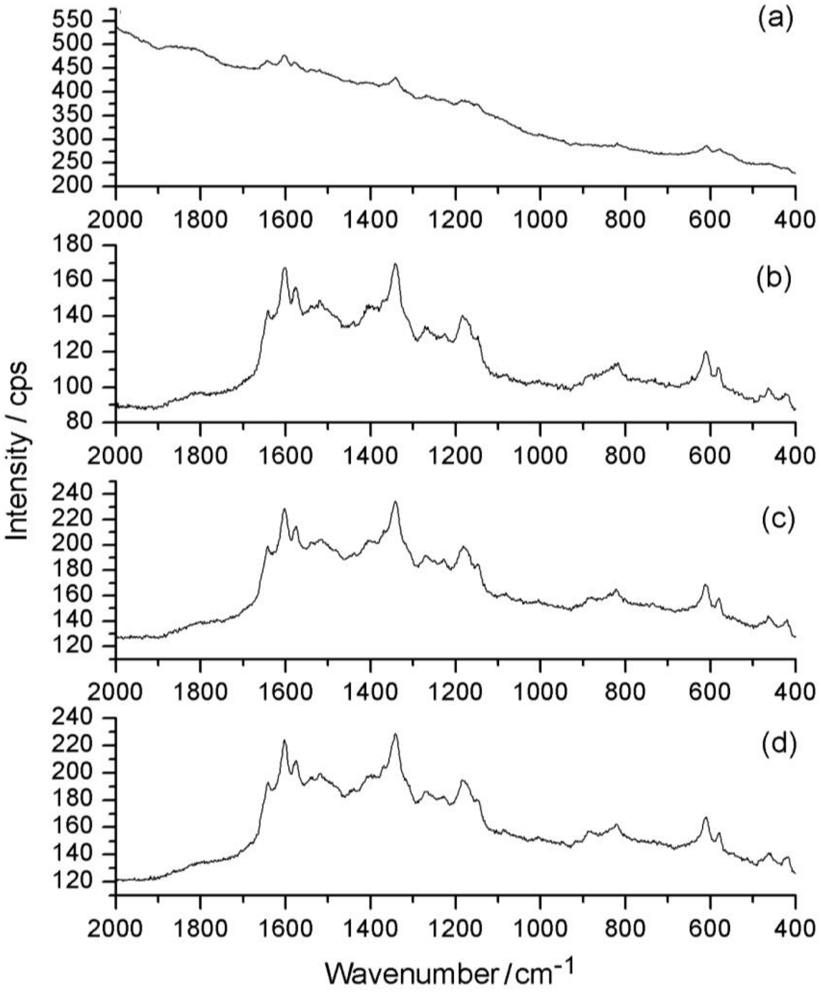

Supplementary Figure S3. *In situ* Raman spectra of the final poly(PADPA)-AOT suspension (recorded ca. 15 months after the beginning of the synthesis) measured using different sample supports: a) glass microscope slide, b) CaF_2 microscope slide, c) Gold EZ-Spot Micro Mount slide and d) Al foil. Excitation wavelength: 633 nm.

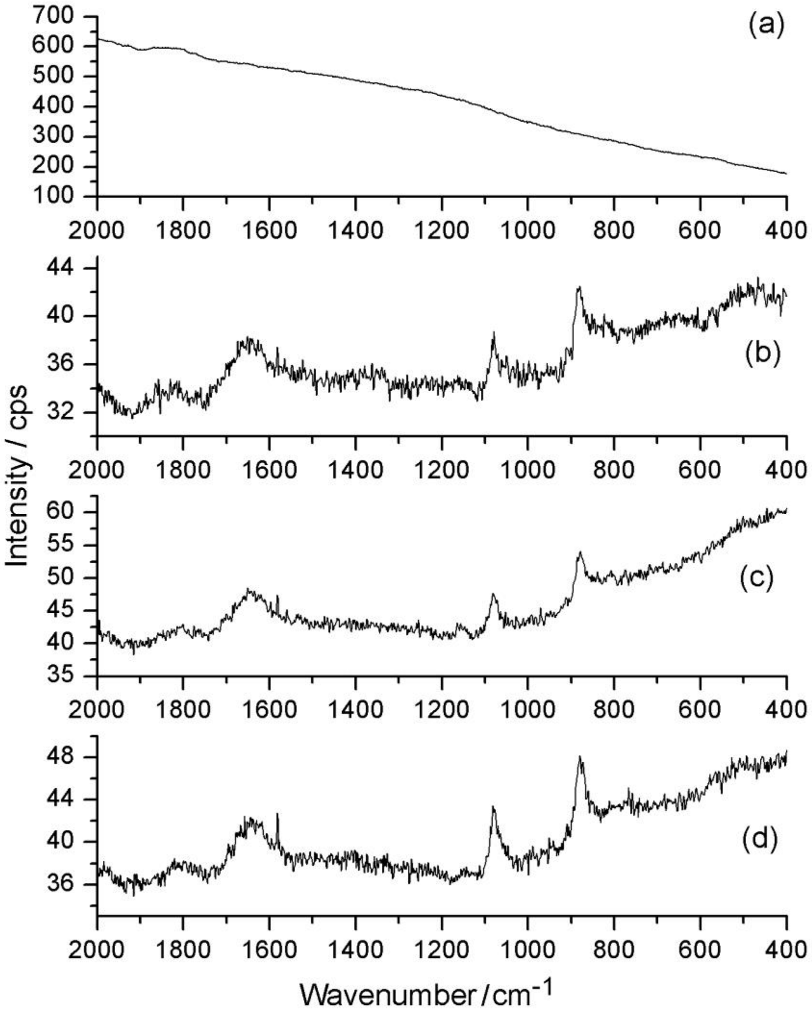

Supplementary Figure S4. Raman spectra of 0.1 M NaH_2PO_4 (aq) measured using different sample supports: a) glass microscope slide, b) CaF_2 microscope slide, c) Gold EZ-Spot Micro Mount slide and d) Al foil. Excitation wavelength: 633 nm.

4. Comparison of the Raman Spectra of Enzymatically Obtained Poly(PADPA) With the Raman Spectra of Chemically Synthesised PANI-ES and PANI-EB

For a direct comparison of the Raman spectra of previously chemically syntheisized PANI-ES products[S8] with the *in situ* Raman spectrum of poly(PADPA) obtained enzymatically with the vesicles, the corresponding spectra are plotted in one single figure (**Supplementary Fig. S5**). Although such comparison may be to some extent limited due to differences in Raman experimental setting (*i.e.*, usage of suspension instead of solid sample and/or the influence of the gold support on the band intensities[S9]), the following conclusions can be drawn at this stage. The *in situ* Raman spectra of the products formed in the presence of AOT vesicles at

longer reaction times (t = 1 day and longer, **Supplementary Fig. S5**) are quite similar to – but by no means identical with − the Raman spectrum of solid, protonated PANI-ES[S8], synthesized by the chemical oxidative polymerization of aniline with ammonium peroxydisulfate (APS) as oxidant in aqueous solution of a strong dopant acid at pH = 1.7 (**Supplementary Fig. S5**). An even greater similarity can be observed if the Raman spectra of poly(PADPA) synthesized in the presence of vesicles at t = 1 day and longer are compared with the spectrum of PANI-ES produced by the classical chemical method at the same initial pH of 3.5 (**Supplementary Fig. S5**); for example, the band at ≈1620 cm^{-1} is present in all spectra. It can be seen that the spectrum of poly(PADPA) synthesized in the presence of vesicles (t = 3 day, **Supplementary Fig. S5**) is more complex than the spectra of both PANI-ES samples, showing additional bands at 1453, 1381, 1360, and 1194 cm^{-1}. This feature indicates that poly(PADPA) synthesized with TvL/O_2 in the presence of vesicles contains certain segments which are different from ordinary PANI-ES segments. For later times, 18 and 38 days, the relative intensity of these additional bands decreases and the spectrum of poly(PADPA) becomes even more similar to the spectrum of PANI-ES (**Fig. 4** and **Supplementary Fig. S5**). On the other hand, the spectra of the products formed without vesicles (t = 1 day and longer, **Fig. 5**) are very similar to the spectrum of solid PANI in the form of the emeraldine base (PANI-EB), **Supplementary Fig. S6**, which is a strong indication of their low conductivity. *In situ* Raman spectroscopy thus supports previous findings[S1] that AOT vesicles promote the formation of delocalized polaron structures and PANI-ES-like molecules during the oxidative polymerization of PADPA with TvL/O_2.

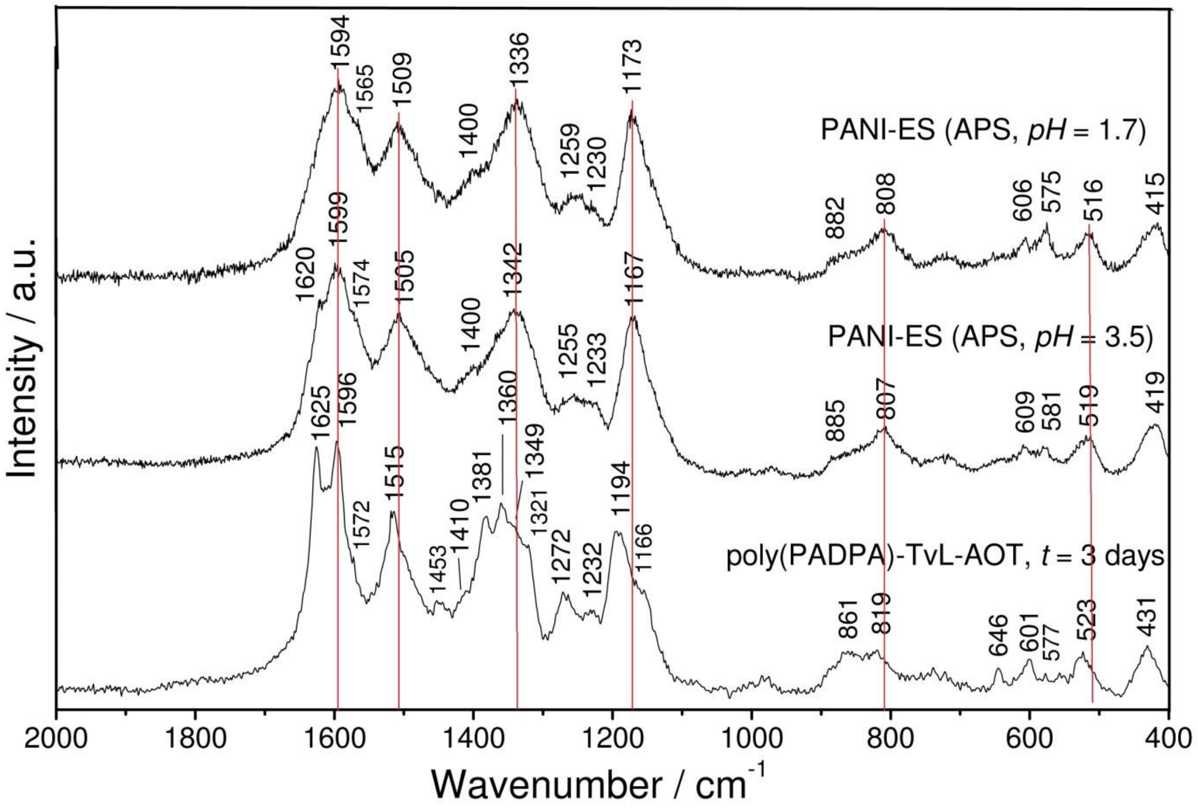

Supplementary Figure S5. Comparison of the *in situ* Raman spectrum of final suspension of poly(PADPA) obtained with TvL/O_2 in the *presence* of AOT vesicles (bottom spectrum) with the Raman spectra of two solid PANI samples in salt forms, prepared chemically by the oxidation of aniline with APS in aqueous solutions of 5-sulfosalicylic acid (SSA) at initial *pH* values of 1.7 and 3.5, *i.e.*, at mole ratios SSA/aniline of 1 and 0.5, respectively (middle and top PANI-ES spectra, taken from Janošević *et al.*, 2008)[S8]. The spectrum of poly(PADPA) is the one shown in **Fig. 4** for *t* = 3 days (fluorescence background corrections were performed). Excitation wavelength for all spectra: 633 nm.

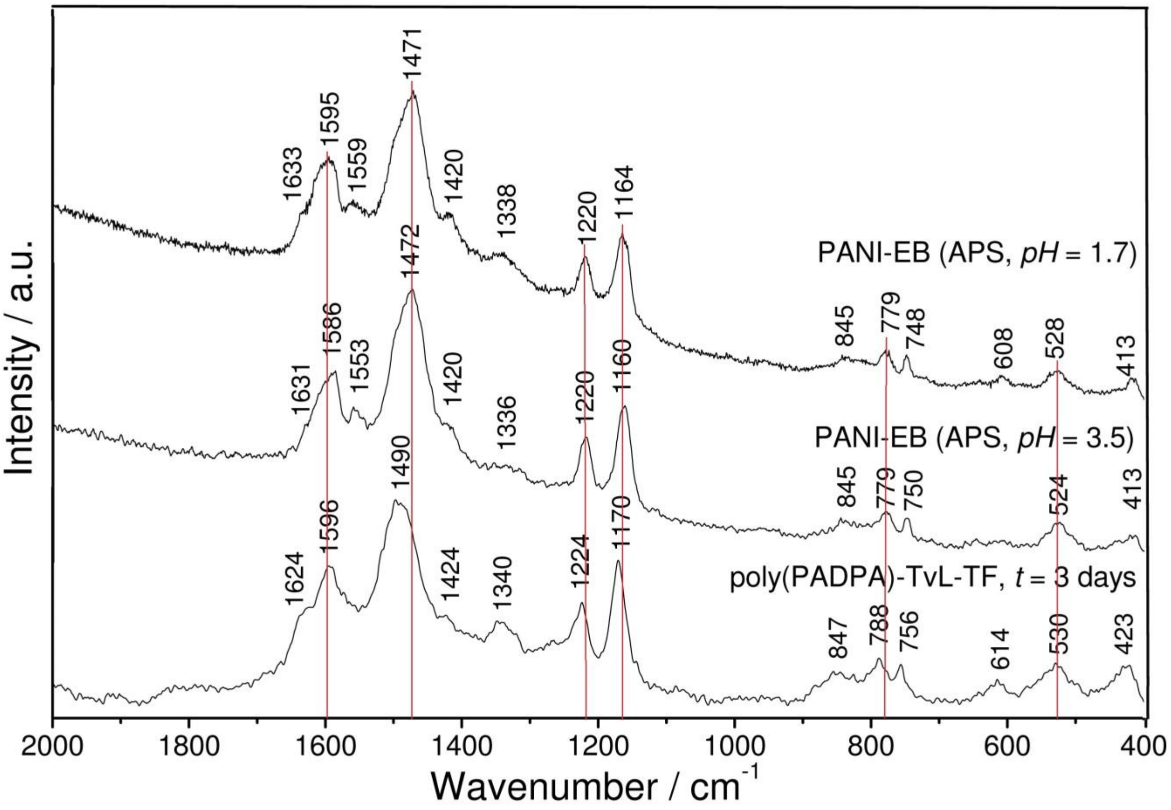

Supplementary Figure S6. Comparison of the *in situ* Raman spectrum of final suspension of poly(PADPA) obtained with TvL/O_2 in the *absence* of vesicles (bottom spectrum) with the Raman spectra of two solid PANI samples in base (deprotonated) forms, synthesized chemically by the oxidation of aniline with APS in aqueous solutions of 5-sulfosalicylic acid (SSA) at initial *pH* values of 1.7 and 3.5, *i.e.* at mole ratios SSA/aniline of 1 and 0.5, respectively (middle and top PANI-EB spectra, Janošević *et al.*, unpublished). The spectrum of poly(PADPA) is the one shown in **Fig. 5** for *t* = 3 days (fluorescence background corrections were performed). Excitation wavelength for all spectra: 633 nm.

5. Raman Spectroscopy Measurements of Poly(PADPA) Isolated from the Reaction Mixtures

The Raman spectra of *solid* poly(PADPA) samples isolated from the reaction mixtures with and without AOT vesicles and purified (abbreviated as poly(PADPA)-AOT and poly(PADPA)-TF), respectively, were also recorded (**Supplementary Fig. S7)**. These spectra were compared mutually and with their counterpart spectra taken *in situ* (**Figs. 4 and 5).**

As a general observation, there are substantial differences between the Raman spectra of *solid* poly(PADPA) and the corresponding *in situ* Raman spectra, both for the vesicle system (spectrum a in **Supplementary Fig. S7;** spectra for *t* >1 day in **Fig. 4**), as well as for the template-free system (spectrum b in **Supplementary Fig. S7**; spectra for *t* > 1 day in **Fig. 5**). These differences most likely indicate that chemical changes occur in poly(PADPA) during workup and product isolation, similar to the observed changes in chemically synthesized PANI-ES during a prolonged heat treatment[S10]. Nevertheless, Raman bands characteristic for PANI-ES-like segments are observed in the Raman spectra of both solid poly(PADPA)-AOT and poly(PADPA)-TF samples at wavenumbers ≈1623 cm^{-1} (shoulder, C~C stretching of B rings, ν(C~C)$_B$), at 1596 cm^{-1} (ν(C~C)$_{SQ}$ / ν(C=C)$_Q$), at 1512 cm^{-1} (δ(N–H), associated with SQ structures), at 1339 cm^{-1} (ν(C–N$^{\bullet+}$) in polaronic structures), at 1177 cm^{-1}, and at 1168 cm^{-1} (δ(C–H)$_{SQ}$ and δ(C–H)$_B$). The bands attributed to B ring deformations are observed at 607 cm^{-1} and 598 cm^{-1} for poly(PADPA)-TF and poly(PADPA)-AOT, respectively[S11,S12]. The bands attributed to ν(C–N)$_B$ vibration are seen at 1240 cm^{-1} and 1230 cm^{-1} for poly(PADPA)-AOT and at 1234 cm^{-1} and 1220 cm^{-1} for poly(PADPA)-TF[S12]. In the spectrum of solid poly(PADPA)-AOT, the presence of quinonediimine units in the sample is indicated by the bands at 1499 cm^{-1} and 1478 cm^{-1} which are attributed to ν(C=N)$_Q$ vibrations (first band being stronger), and by the weak band at 1152 cm^{-1} due to δ(C–H)$_Q$ vibrations[S12]. The appearance of two ν(C=N)$_Q$ bands indicates the presence of two components in solid poly(PADPA)-AOT sample bearing different Q-type rings. The spectrum of solid poly(PADPA)-TF shows only one ν(C=N)$_Q$ band at 1497 cm^{-1}, which has a much higher relative intensity than the corresponding band at 1499 cm^{-1} in the spectrum of poly(PADPA)-AOT (**Supplementary Fig. S7**). Since the high conductivity of PANI-type polymers is widely accepted to originate from delocalized polarons as charge carriers, the last feature indicates lower conductivity of poly(PADPA)-TF compared to poly(PADPA)-AOT. Qualitatively, there is agreement with the findings obtained from *in situ* Raman measurements, confirming that AOT vesicles act in favor of the formation of PANI-ES-like products with good charge mobility (electrical conductivity).

An additional important observation is that „the polaron band" at 1339 cm^{-1} is stronger than the bands attributable to phenazine-type units (at wavenumbers 1570, 1409, and 1391/1399 cm^{-1}) for solid poly(PADPA)-AOT than for poly(PADPA)-TF. Also, the intensity ratio of „the polaron band" at 1339 cm^{-1} to the intensity of the ν(C=N)$_Q$ band at 1497 cm^{-1} is significantly higher for poly(PADPA)-AOT than for poly(PADPA)-TF. These spectral

characteristics also indicate a higher charge mobility, *i.e.*, a higher conductivity, of the solid products formed in the presence of vesicles as templates if compared to the products obtained without vesicles.

The main observations regarding the differences between the Raman spectra of isolated poly(PADPA) samples and corresponding *in* situ measured Raman spectra of poly(PADPA) products are summarized and discussed in the following. Besides the bands attributable to PANI-ES-like segments, several additional bands are observed in the Raman spectra of solid poly(PADPA)-AOT and poly(PADPA)-TF samples (**Supplementary Fig. S7**) which can be correlated with the segments which are different from those of ordinary PANI-ES („non-ordinary" segments). The presence of phenazine-, *N*-phenylphenazine- and/or phenoxazine-type segments in poly(PADPA)-AOT and poly(PADPA)-TF is indicated by the bands at 1648, 1639, 1583, 1570, 1530, 1409, 1391, 575 cm^{-1} and at 1570, 1542, 1407, 1407, 1399, 576 cm^{-1}, respectively (**Supplementary Fig. S7**)[S12]. These types of segments may be produced by the oxidative intramolecular cyclization of branched units present in the formed products[S11,S12,S13,S14]. The band at ca. 575 cm^{-1} is attributable to phenoxazine-type units[S11,S12] and is much stronger in the spectrum of poly(PADPA)-AOT. It is interesting to note that the *in situ* Raman spectra recorded for the final products formed in the presence of AOT vesicles (**Fig. 4**) do not contain bands of „non-ordinary" segments at 1648, 1530, and 575 cm^{-1}, and, similarly, the *in situ* Raman spectra of products formed in the absence of vesicles (**Fig. 5**) do not show the bands at 1542 and 576 cm^{-1}. All in all, it seems that the appearance of these bands in the spectra of solid poly(PADPA) samples is caused by the isolation and/or purification procedure which leads to some chemical changes in the products. This is an important finding and must be kept in mind if other spectroscopic data from in situ measurements are compared with spectroscopic data of solid products, *e.g.*, EPR measurements[S15]. In order to keep the chemical structure of the actually obtained poly(PADPA) product intact, it is better to directly use the as-obtained poly(PADPA)-AOT products. This is possible since the poly(PADPA)-AOT vesicle suspension is of high colloidal stability and can be applied directly, as shown below in the case of cyclic voltammetry measurements.

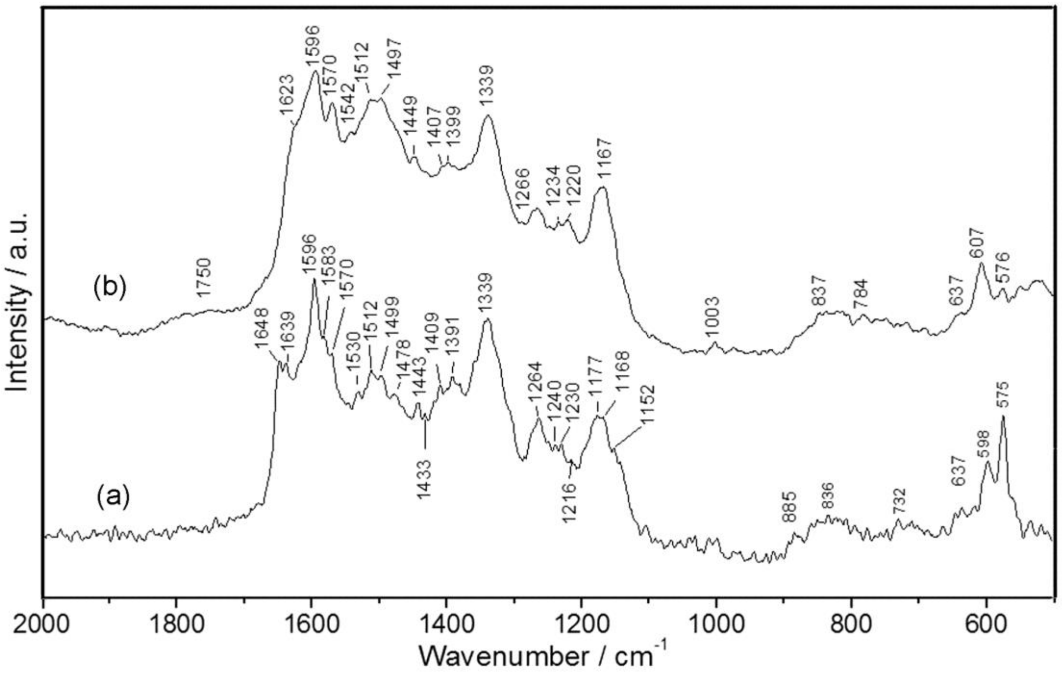

Supplementary Figure S7: Raman spectra of isolated and purified solid poly(PADPA) samples synthesized by the polymerization of PADPA with TvL/O_2 in the presence of AOT vesicles (a) and without vesicles (b), see *Methods*. Excitation wavelength: 633 nm. The spectra were obtained after fluorescence background corrections.

6. Comment on the Sample Preparation for the Cyclic Voltammetry Measurements

The current responses of poly(PADPA) deposited using the thin layer and direct drop-cast approaches are different from each other due to different masses of the electroactive species on the electrode and due to a possible influence of the isolation/purification procedure on the molecular structure of poly(PADPA) (as indicated by the Raman spectroscopic analysis, see **Supplementary Fig. S7**). However, their current peaks appear to be distributed in a similar fashion. Nevertheless, the voltammograms of poly(PADPA) obtained using the thin layer approach are tilted with the current peaks being slightly distorted. This is most likely due to the charge transfer resistance through the thin film on the electrode and due to the capacitive response of Vulcan XC-72R added as a current collector (see *Methods*) which is superimposed on the faradic response of poly(PADPA).

Direct drop-casting of the reaction mixture provides a simple and effective route for the preparation of a film of poly(PADPA) on the electrode surface. This enables a direct evaluation of the redox properties of the products without the risk of post-polymerization changes during the isolation and/or purification as was found by using Raman spectroscopy (see **Supplementary Fig. S7**).

7. Set-up for the *in situ* Raman Monitoring

We found the usage of Gold EZ-Spot Micro Mount as a sample platform especially suitable for the *in situ* Raman measurements of the investigated systems in the present work due to the following advantages. Gold EZ-Spot Micro Mount is a 25 mm × 75 mm × 1 mm sample slide with 12 sample wells (mounts), each with a diameter of 4 mm. This is a gold-coated glass slide with a polytetrafluoroethylene (Teflon) top layer (thickness 0.05 mm). Circular indentations on this layer form 12 wells on the gold surface of the slide (**Supplementary Fig. S8)**. The wells thus serve as 'microreactors' with a gold bottom and Teflon walls (both materials being chemically inert) into which we transferred small aliquots (5 µL volume) of the reaction mixture from the main reaction vessel, at different times of reaction (**Supplementary Fig. S8**). Thanks to the precisely defined sample wells, flowing and spilling of the sample (*e.g.* during manipulation and transportation to the instrument) are avoided. This enables reproducible measurements and facilitates manual focusing of the Raman microscope required before recording the spectrum, which is especially important when the time between two successive measurements of the spectra during the reaction is short. Compared with optical fibre immersion probe technique which is commonly used for *in situ* Raman measurements, the here applied technique has no problem with a time-to-time required cleaning of the immersion probe head surface to remove adsorbed products, and, in addition, this technique can be used for the experiments with very small reaction volumes.

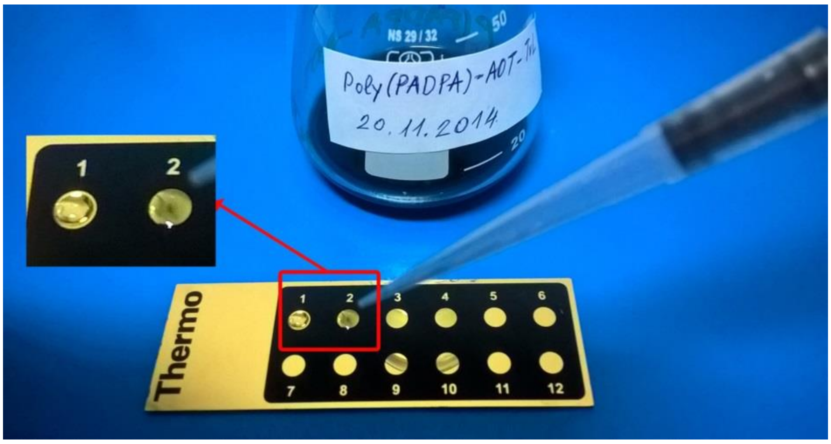

Supplementary Fig. S8. Photograph of Gold EZ-Spot Micro Mount slide with 12 sample wells: 5 µL of 0.1 M NaH_2PO_4 is placed into the well No. 1 and 5 µL of the reaction mixture containing poly(PADPA) synthesized with TvL/O_2 in the presence of AOT vesicles is taken from the reaction vessel and placed into the well No. 2.

8. References

(S1) Junker, K. *et al.* Efficient polymerization of the aniline dimer *p*–aminodiphenylamine (PADPA) with *Trametes versicolor* laccase/O_2 as catalyst and oxidant and AOT vesicles as templates. *ACS Catal.* **4,** 3421−3434 (2014).

(S2) Žilić, D. *et al.* Single crystals of DPPH grown from diethyl ether and carbon disulfide solutions − crystal structures, IR, EPR and magnetization studies. *J. Magn. Reson.* **207,** 34−41 (2010).

(S3) Walrafen, G. E. Raman spectral studies of the effects of electrolytes on water. *J. Chem. Phys.* **36,** 1035−1042 (1962).

(S4) Socrates, G. *Infrared and Raman Characteristic Group Frequencies*, *3rd edn* (John Wiley & Sons, 2001).

(S5) Marshall, W. L. & Begun, G. M. Raman spectroscopy of aqueous phosphate solutions at temperatures up to 450 °C. *J. Chem. Soc., Faraday Trans. 2* **85,** 1963−1978 (1989).

(S6) Nagasoe, Y. *et al.* Raman and IR spectroscopic studies of the interaction between counterion and polar group in self-assembled systems of AOT-homologous "sodium dialkyl sulfosuccinates". *Phys. Chem. Chem. Phys.* **1,** 4395−4407 (1999).

(S7) Arunagirinathan, M. A., Roy, M., Dua, A. K., Manohar, C. & Bellare, J. R. Micro-Raman investigations of myelins in Aerosol-OT/water system. *Langmuir* **20,** 4816−4822 (2004).

(S8) Janošević, A. *et al.* Synthesis and characterization of conducting polyaniline 5-sulfosalicylate nanotubes. *Nanotechnology* **19,** 135606 (8pp) (2008).

(S9) Trchová, M., Morávková, Z., Dybal, J. & Stejskal, J. Detection of aniline oligomers on polyaniline−gold interface using resonance Raman scattering. *ACS Appl. Mater. Interfaces* **6,** 942−950 (2014).

(S10) Sěděnková, I.; Trchová. M.; Stejskal, J. Thermal Degradation of Polyaniline Films Prepared in Solutions of Strong and Weak Acids and in Water – FTIR and Raman Spectroscopic Studies. *Polymer Degrad. Stab.* **2008**, *93*, 2147−2157.

(S11) Ćirić-Marjanović, G., Trchová, M., Konyushenko, E. N., Holler, P. & Stejskal, J. Chemical oxidative polymerization of aminodiphenylamines. *J. Phys. Chem. B* **112,** 6976−6987 (2008).

(S12) Ćirić-Marjanović, G., Trchová, M. & Stejskal, J. The chemical oxidative polymerization of aniline in water: Raman spectroscopy. *J. Raman Spectrosc.* **39,** 1375−1387 (2008).

(S13) Ćirić-Marjanović, G., Trchová, M. & Stejskal, J. Theoretical study of the oxidative polymerization of aniline with peroxydisulfate: Tetramer formation. *Int. J. Quantum Chem.* **108,** 318−333 (2008).

(S14) Ćirić-Marjanović, G., Trchová, M. & Stejskal, J. MNDO-PM3 study of the early stages of the chemical oxidative polymerization of aniline. *Collect. Czech. Chem. Commun*. **71,** 1407−1426 (2006).

(S15) Carić, D. *et al.* Multifrequency EPR study of poly(PADPA) synthesized with *Trametes versicolor* laccase from the aniline dimer *p*-aminodiphenylamine (PADPA) in the presence of anionic vesicles. *Curr. Appl. Phys.* **15,** 1516−1520 (2015).
